# TCR-mimic bispecific nanobody-based T cell engager targeting intracellular tumor antigens for cancer immunotherapy

**DOI:** 10.1038/s41392-026-02745-x

**Published:** 2026-06-25

**Authors:** Ziqiang Ding, Shuyang Sun, Xiaomei Yang, Xianing Huang, Xiaoqiong Hou, Shenxia Xie, Aiqun Liu, Xiaoling Lu

**Affiliations:** 1 School of Basic Medical Sciences/College of Stomatology/Hospital of Stomatology/Guangxi Key Laboratory of Nanobody Research/Guangxi Nanobody Engineering Research Center, Guangxi Medical University, Nanning, China; 2 Department of Laboratory Medicine, Nanjing Drum Tower Hospital, Affiliated Hospital of Medical School, Nanjing University, Nanjing, China

**Keywords:** Tumour immunology, Immunotherapy

## Abstract

T cell engager (TCE) immunotherapies have revolutionized the landscape of cancer treatment; however, their efficacy remains limited by the inaccessibility of intracellular tumor antigens. Conventional bispecific T cell engagers, typically constructed from aggregation-prone single-chain variable fragments (scFvs), suffer from structural instability and an antigenic scope restricted to extracellular targets. To overcome these critical limitations, we presented a proof-of-concept study establishing a modular bispecific VHH‑VHH immunotherapeutic platform. Specifically, we developed a first-in-class TCR-mimic bispecific nanobody (Nb)-based T cell engager (TCRm Bi-NbTE) platform that simultaneously engages CD3ε on T cells and tumor-specific peptide-MHC class I (pMHC I) complexes, exemplified by HLA-A2/WT1_126-134_ or HLA-A2/GPC3_144-152_. Functional analyses in vitro and in vivo studies demonstrated that TCRm Bi-NbTE exhibits exceptional specificity, potently induces antigen-restricted T cell activation, and mediates selective lysis of pMHC I⁺ tumor cells while sparing antigen-negative cells. In multiple mouse xenograft models, including both cell-derived xenograft (CDX) and patient-derived xenograft (PDX) models, TCRm Bi-NbTE significantly suppressed tumor growth, prolonged survival, and enhanced T cell infiltration without treatment-related adverse effects. By redirecting T cell against intracellular antigens in an HLA-restricted manner, TCRm Bi-NbTE establishes a modular, scalable, and clinically translatable platform for next-generation cancer immunotherapy across a broad spectrum of solid and hematologic malignancies.

## Introduction

T cell-based immunotherapies have revolutionized tumor treatment by harnessing the body’s immune system to eradicate malignant cells, and strategies are under investigation to redirect T cells to recognize tumor cells.^1–3^ Among these, BiTE^®^ (bispecific T cell engager) is an engineered antibody that simultaneously binds to CD3 on T cells and tumor-associated antigens (TAAs) on tumor cells, facilitating efficient T cell-mediated cytotoxicity.^4,5^ The clinical success of blinatumomab®, a CD19/CD3 BiTE approved for B-cell malignancies, has validated the therapeutic potential of BiTE molecules in hematological cancers.^6–8^ Despite remarkable advances, conventional single-chain variable fragment (scFv)-based BiTE construct suffer from structural instability, low solubility, and aggregation propensity, leading to technical challenges during production and restricted clinical translation.^9,10^ Furthermore, BiTE molecules predominantly target extracellular TAAs, which represent less than 30% of known tumor-specific proteins, while the vast majority of tumor antigens are intracellular or secreted,^11–13^ rendering them inaccessible to conventional BiTE therapies.

Recent developments in T cell engager engineering have introduced nanobodies (Nbs), which are variable VHH domains derived from camelid heavy-chain-only antibodies, as promising alternatives to scFvs.^10,14^ Nbs possess a compact size, high affinity, superior tissue penetration and facile genetic engineering properties.^15,16^ Importantly, Nbs circumvent VH-VL domain mispairing inherent in multi-chain antibodies, enabling versatile and scalable engineering for next-generation T cell engagers.^17^ Our previous work developed a nanobody-based bi- and tri-specific T cell engager platform that demonstrated potent antitumor activity in preclinical models.^18,19^ However, its reliance on cell surface antigens limited therapeutic applicability and raised concerns regarding on-target/off-tumor toxicity due to antigen expression in certain normal tissues. Therefore, to overcome these barriers and broaden the spectrum of targetable tumor antigens, innovative immunotherapy in order to augment the efficacy of nanobody-based T cell engager strategies are needed.

Most cellular proteins are processed by the proteasome into peptides presented on major histocompatibility complexes (MHC) class I molecules as peptide-MHC (pMHC) complexes, which are recognized by T cell receptors (TCRs) on CD8^+^ cytotoxic T cells to initiate immune responses.^20,21^ Among tumor-associated antigens presented via pMHC, Wilms tumor 1 (WT1) and glypican-3 (GPC3) represent ideal targets due to their high expression in certain solid tumors or hematological malignancies while exhibiting minimal expression in normal tissues.^22–26^ To exploit these intracellular antigens, TCR-mimic (TCRm) antibodies have been developed to target these intracellular antigens presented as pMHC complexes.^27,28^ Our group previously generated TCRm nanobodies specific for HLA-A2/WT1_126-134_ and HLA-A2/GPC3_144-152_ complexes, which demonstrated selective and effective targeting.^26^ Nevertheless, the efficacy of TCRm-based therapies is often constrained by the relatively low epitope density of pMHC complexes on tumor cells. Early-generation ESK1-BiTE have shown promising tumor-specific cytotoxicity in preclinical studies,^23^ underscoring the potential of integrating TCRm specificity into a nanobody-engineered T cell-engaging framework.

Here, we present a proof-of-concept study establishing a modular bispecific VHH-VHH immunotherapeutic platform that broadens the therapeutic applicability of TCRm-based therapies by engineering a novel TCRm bispecifc nanobody-based T cell engager (TCRm Bi-NbTE). This design enhances tumor selectivity through dual recognition of both HLA-A2 and the specific tumor peptides derived from a broader spectrum of intracellular antigens. Specifically, this TCRm Bi-NbTE construct combines two Nbs, one targeting CD3ε on T cells and the other directed against a tumor-specific pMHC complex on tumor cells, thereby enabling redirection of T cells to intracellular tumor antigens independent of their endogenous TCR specificity. The resulting TCRm Bi-NbTE molecule facilitates efficient T cell-tumor cell engagement, enhances T cell activation, and mediates antigen-restricted cytotoxicity, thereby provides a versatile platform adaptable to other types tumor-associated antigens.

In this study, to exemplify the novel TCRm Bi-NbTE, we present the generation and therapeutic characterization of TCRm Bi-NbTE molecules targeting the HLA-A2/WT1_126-134_ and HLA-A2/GPC3_144-152_ complexes. Using models of HLA-A2^+^/WT1^+^ and HLA-A2^+^/GPC3^+^ targets, we demonstrated that TCRm Bi-NbTE effectively redirects T cells to specifically lyse pMHC^+^ tumor cells, while sparing antigen-negative cells lacking the antigenic complex. Meanwhile, TCRm Bi-NbTE enhances immune signaling connection, promotes T cell activation and proliferation, and elicits potent antitumor activity both in vitro and in vivo. Collectively, this work represents the first attempt to integrate TCRm nanobody specificity within a fully nanobody-based T cell engager framework. The resulting TCRm Bi-NbTE offers a universal, modular and scalable immunotherapeutic platform capable of unlocking the intracellular tumor antigens for next-generation cancer immunotherapy across the spectrum of malignancies.

## Results

### Design and identification of TCRm Bi-NbTE

**Fig. 1:**
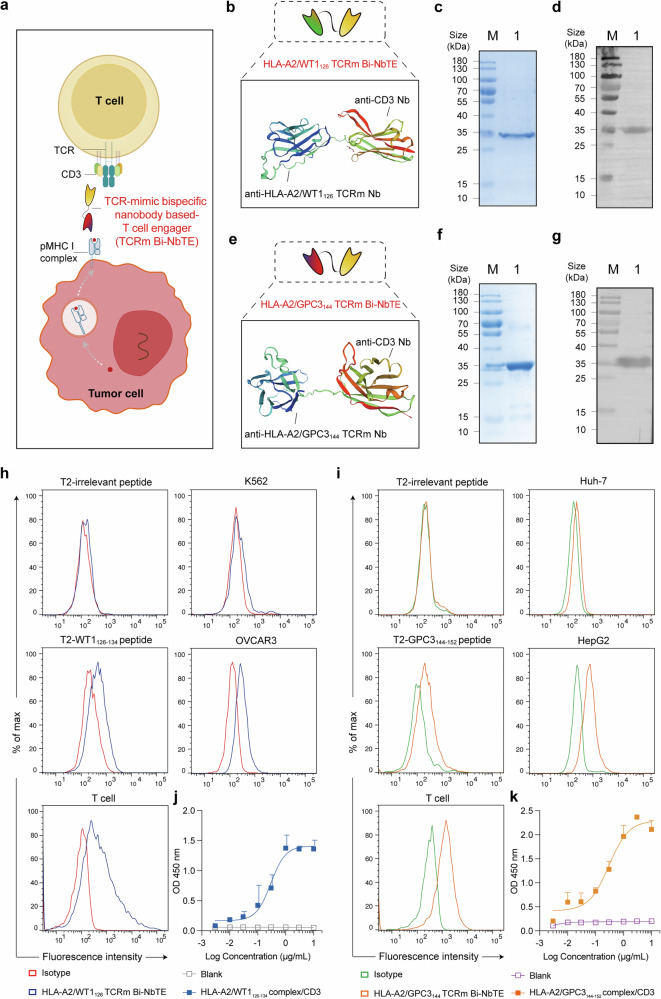
Generation and characterization of TCRm Bi-NbTE. **a** Schematic diagram of the TCRm Bi-NbTE molecule, consisting of a TCRm nanobody targeting pMHC complex and a CD3ε nanobody engaging T cells. Predicted structural models of (**b**) HLA-A2/WT1_126_ TCRm Bi-NbTE and (**e**) HLA-A2/GPC3_144_ TCRm Bi-NbTE were generated using the SWISS-MODEL model (https://swissmodel.expasy.org). SDS–PAGE analysis of purified (**c**) HLA-A2/WT1_126_ TCRm Bi-NbTE and (**f**) HLA-A2/GPC3_144_ TCRm Bi-NbTE was visualized by Coomassie blue staining. Lanes: M, marker; 1, purified protein. Western blot validation of purified (**d**) HLA-A2/WT1_126_ TCRm Bi-NbTE and (**g**) HLA-A2/GPC3_144_ TCRm Bi-NbTE using an anti-His tag antibody. Lanes: M, marker; 1, purified protein. **h** Binding analysis of HLA-A2/WT1_126_ TCRm Bi-NbTE to HLA-A2^+^/WT1^+^ OVCAR3 cells, HLA-A2^-^/WT1^+^ K562 cells, T2 cells pulsed with WT1_126-134_ peptides or irrelevant peptides, and primary human T cells assessed by flow cytometry. **i** Binding analysis of HLA-A2/GPC3_144_ TCRm Bi-NbTE to HLA-A2^+^/GPC3^+^ HepG2 cells, HLA-A2^-^/GPC3^+^ Huh-7 cells, T2 cells pulsed with GPC3_144-152_ peptides or irrelevant peptides, and primary human T cells assessed by flow cytometry. **j**, **k** Sandwich ELISA demonstrating that TCRm Bi-NbTEs simultaneously engage CD3 and their respective peptide-MHC complexes without steric interference

To enable the redirection of T cell against intracellular tumor antigens in a WT1^+^ or GPC3^+^ and HLA-A2^+^ restricted manner, we engineered a novel TCRm Bi-NbTE antibody. Specifically, an anti-CD3ε nanobody previously screened and characterized was fused via a flexible Gly_4_Ser linker to a TCRm nanobody targeting either the HLA-A2/WT1_126-134_ complexes or HLA-A2/GPC3_144-152_ complexes, both identified in our earlier studies (Fig. 1a). A negative control construct (Irrelevant NbTE) was generated by replacing the TCRm nanobody with an irrelevant nanobody derived from the human antibody germline repertoire that recognizes a nontumor target. Protein homology modeling of the designed TCRm Bi-NbTE constructs was performed using the SWISS model (https://swissmodel.expasy.org), yielding a predicted spatial arrangement of the molecules (Fig. 1b, e). While the TCR Bi-NbTE format is distinct from previously reported TCRm bispecific antibodies,^29^ ImmTACs^30^ and TCRm BiTE molecules,^23^ it aligns with the modularity of the nanobody-based bispecific T cell engager format (Supplementary Fig. S1a). Heterologous expression in *E. coli* BL21(DE3) revealed that the HLA-A2/WT1_126_ TCRm Bi-NbTE exhibited maximal yield under optimal induction with 0.5 mM IPTG at 37 °C for 6 h (Supplementary Fig. S2a, c, d), whereas the HLA-A2/GPC3_144_ TCRm Bi-NbTE achieved optimal expression under induction conditions with 0.5 mM IPTG at 16 °C for 16 h (Supplementary Fig. S2b, e, f). Both TCRm Bi-NbTE proteins were efficiently purified from inclusion bodies by a Ni^2+^–NTA affinity chromatography (Supplementary Fig. S3a, b). Purity was confirmed to exceed 90% by SDS–PAGE and SEC–HPLC analysis (Fig. 1c, f, Supplementary Fig. S3c, d). Western blotting with anti-His tag antibodies further verified molecular identities with expected immunoreactive band sizes (Fig. 1d, g). Overall, these results demonstrate the successful generation and purification of functional HLA-A2/WT1_126_ TCRm Bi-NbTE and HLA-A2/GPC3_144_ TCRm Bi-NbTE proteins, establishing a robust foundation for subsequent binding and functional evaluation. This innovative TCRm Bi-NbTE design extends the BiTE therapeutic repertoire for targeting intracellular tumor antigens.

### Binding specificity of TCRm Bi-NbTE

To systematically characterize the binding specificity of TCRm Bi-NbTE, we performed flow cytometry-based binding assays using both antigen-positive and antigen-negative cell lines. The HLA-A2/WT1_126_ TCRm Bi-NbTE specifically bound to OVCAR3 (HLA-A2^+^/WT1^+^) and T2-WT1_126-134_ cells (HLA-A2^+^/WT1^+^), as well as primary human T cells, with no detectable binding to K562 cells (HLA-A2^-^/WT1^+^), T2-irrelevant peptide cells or unpulsed (Fig. 1h, Supplementary Fig. S4a-c). Similarly, the HLA-A2/GPC3_144_ TCRm Bi-NbTE exhibited specific binding to HepG2 (HLA-A2^+^/GPC3^+^) and T2-GPC3_144-152_ (HLA-A2^+^/GPC3^+^) cells, as well as primary human T cells, while no binding was observed for Huh-7 (HLA-A2^-^/GPC3^+^), T2-irrelevant peptide cells or unpulsed (Fig. 1i, Supplementary Fig. S4a, d, e). Importantly, competitive inhibition assays confirmed the binding specificity of TCRm Bi-NbTE. Preincubation of T cells with soluble recombinant hCD3ε protein significantly reduced TCRm Bi-NbTE binding (Supplementary Fig. S4c, e). Furthermore, binding affinity of the TCRm Bi-NbTE was determined by biolayer interferometry (BLI). The HLA-A2/WT1_126_ TCRm Bi-NbTE displayed equilibrium dissociation constants (KD) of 2.49 × 10^−8^ M for recombinant CD3ε and 5.39 × 10^−7^ M for the HLA-A2/WT1_126-134_ complex protein (Supplementary Fig. S5a, b). Similarly, the HLA-A2/GPC3_144_ TCRm Bi-NbTE showed 1.18 × 10^−8^ M for CD3ε and 1.01 × 10^−7^ M for the HLA-A2/GPC3_144-152_ complex protein (Supplementary Fig. S5d, e). No measurable binding was detected toward mimetic peptides (Supplementary Fig. S5c, f). Moreover, a sandwich enzyme‑linked immunosorbent assay (ELISA) results showed that TCRm Bi-NbTE can simultaneously bind to two target antigens, indicating no steric interference between epitopes (Fig. 1j, k). These results establish TCRm Bi-NbTE as a dual-specific and HLA-A2/peptide-restricted T cell engager capable of simultaneously recognizing CD3 and pMHC complexes.

To further assess potential off-target interactions, we evaluated its cross-reactivity using the sCRAP algorithm, which predicts structural similarities between target epitopes and human proteome-derived peptides presented on HLA-A*02:01.^31^ Based on these predictions, four top-ranked homologous peptides were identified and selected for experimental validation.^26^ Flow cytometry analysis revealed that no detectable binding by either TCRm Bi-NbTE to T2 cells pulsed with these predicted cross-reactive peptides (Supplementary Fig. S6a, b). Alanine-scanning further identified the critical contact residues mediating recognition. Specifically, substitutions at Arg1, Asn5, Ala6, and Leu9 significantly diminished HLA-A2/WT1_126_ TCRm Bi-NbTE binding, indicating these residues as critical contribution to recognition. Similarly, residues Val2, Gly3, Phe5, and Val9 are particularly important for recognition by HLA-A2/GPC3_144_ TCRm Bi-NbTE (Supplementary Fig. S6c, d). Taken together, these data confirm high specificity and dual antigen recognition of TCRm Bi-NbTE, supporting its therapeutic potential against intracellular tumor antigens in an HLA-restricted manner.

### TCRm Bi-NbTE triggers T cell activation, proliferation, and effector function

**Fig. 2:**
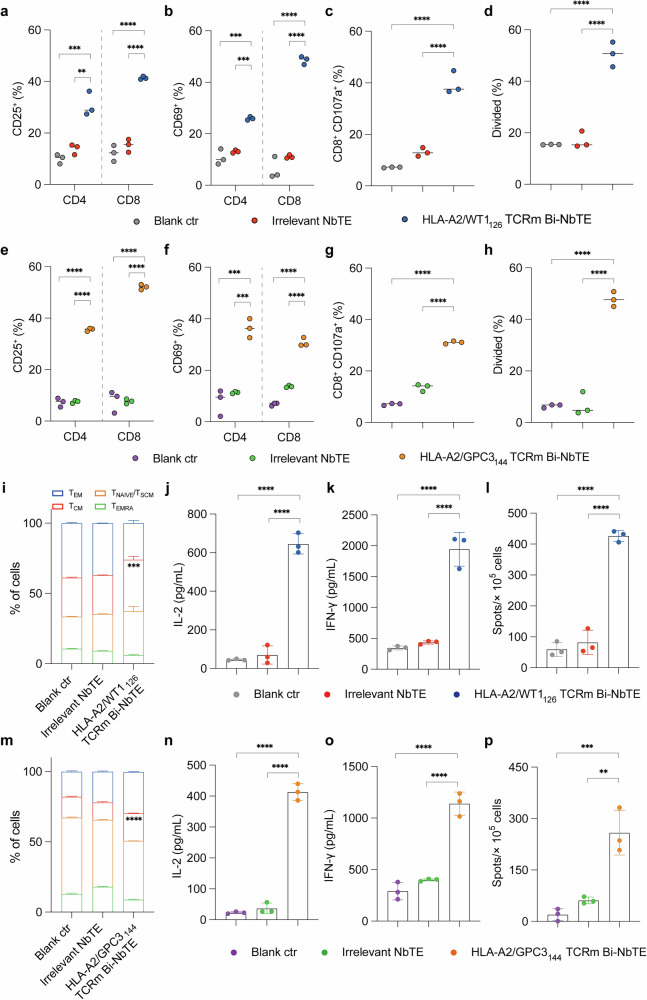
TCRm Bi-NbTE induced T cell activation and effector function. Upregulation of (**a**, **e**) CD25, (**b**, **f**) CD69, and (**c**, **g**) CD107a on T cells after co-culture with OVCAR3 or HepG2 cells in the presence of TCRm Bi-NbTE analyzed by flow cytometry. **d**, **h** T cell proliferation following co-cultured with OVCAR3 cells or HepG2 cells in the presence of respective TCRm Bi-NbTE measured by flow cytometry and presented as division percentage. T cell subpopulation analysis after stimulation with (**i**) HLA-A2/WT1_126_ TCRm Bi-NbTE or (**m**) HLA-A2/GPC3_144_ TCRm Bi-NbTE, defined by CCR7 and CD45RA expression. **j**, **n** IL-2 and **k**, **o** IFN-γ secretion levels in co-cultures of T cells with OVCAR3 cells or HepG2 cells in the presence of TCRm Bi-NbTE or at equimolar controls. ELISPOT assays quantifying IFN-γ-secreting T cells induced by (**l**) HLA-A2/WT1_126_ TCRm Bi-NbTE or (**p**) HLA-A2/GPC3_144_ TCRm Bi-NbTE. Data are representative of three independent experiments. **P < 0.01; ***P < 0.001; ****P < 0.0001

To assess the functional responses of TCRm Bi-NbTE-mediated T cell engagement, we analyzed T cell activation, proliferation and cytokine production when co-cultures with OVCAR3 (HLA-A2^+^/WT1^+^) or HepG2 (HLA-A2^+^/GPC3^+^) cells. Strikingly, compared to Irrelevant NbTE or blank controls (ctr), both TCRm Bi-NbTE induced robust T cell activation, evidenced by significantly upregulated surface expression of CD25 (Fig. 2a, e, Supplementary Fig. S7a, c) and CD69 (Fig. 2b, f, Supplementary Fig. S7a, c). Concurrently, degranulation activity, measured by CD107a expression increased in TCRm Bi-NbTE-mediated T cells compared to Irrelevant NbTE or blank ctr (Fig. 2c, g, Supplementary Fig. S7a, c). Meanwhile, no T cell activation was observed when TCRm Bi-NbTE was applied to both HLA-A2⁺/antigen⁻ tumor cell lines (ARH77, SW480) or normal CD34^+^ cells from an HLA-A2^+^ healthy donor (Supplementary Fig. S9a-c), further supporting that TCRm Bi-NbTE triggering potent antigen-dependent T cell responses.

Another key hallmark of activated T cell is their proliferative capacity. Flow cytometry analysis demonstrated that TCRm Bi-NbTE mediated substantial T cell proliferation in pMHC^+^ tumor co-cultures, whereas Irrelevant NbTE or blank ctr exhibited minimal cell division (Fig. 2d, h, Supplementary Fig S7a, c). Notably, TCRm Bi-NbTE also promoted differentiation into central memory T cells (TCMs, CD45RA^−^/CCR7^+^), increasing their proportion compared to controls (Fig. 2i, m, Supplementary Fig. S7b, d). This phenotype is associated with heightened proliferative potential, lymphoid homing and rapid effector differentiation upon antigen re-encounter, suggesting enhanced potential for sustained antitumor immunity.

Consistent with this activated state, TCRm Bi-NbTE engagement triggered a potent antigen-specific cytokine response. Secretion of key effector cytokines, including IL-2 and IFN-γ, was significantly elevated compared to Irrelevant NbTE in the presence of pMHC^+^ tumor cells (Fig. 2j, k, n, o). Enzyme-linked immunospot (ELISPOT) analysis revealed an increase in IFN-γ^+^ T cell frequencies in TCRm Bi-NbTE-treated groups (Fig. 2l, p). Taken together, these data demonstrate that TCRm Bi-NbTE selectively activate and expand T cells in an antigen-dependent manner, driving potent effector function, align with emerging evidence highlighting the therapeutic value of antigen-restricted T cell engagers in cancer immunotherapy.

### TCRm Bi-NbTE mediates specific cytotoxicity against pMHC^+^ tumor cells in vitro

**Fig. 3:**
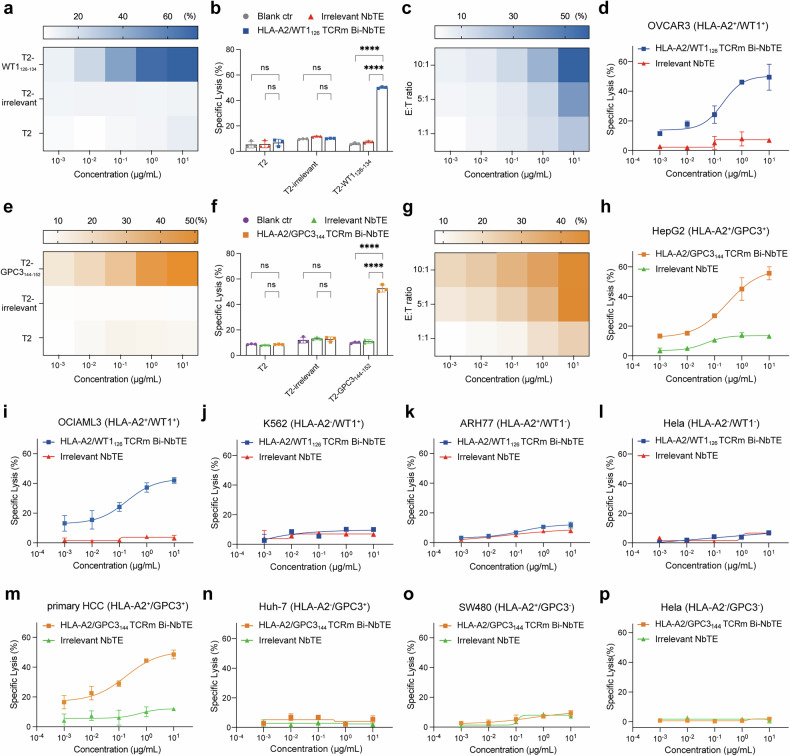
TCRm Bi-NbTE mediated T cell-specific cytotoxicity against pMHC^+^ complex target cells in vitro. Dose-dependent cytotoxicity of T2 cells pulsed with (**a**) WT1_126-134_ peptide or (**e**) GPC3_144-152_ peptide in the presence of various concentrations of TCRm Bi-NbTE, and specific lysis of T2 cells pulsed with (**b**) WT1_126-134_ peptide or (**f**) GPC3_144-152_ peptide at E:T ratio of 10:1 compared to T2 cells pulsed with irrelevant peptide, or unpulsed. E:T ratio-dependent cytotoxicity against (**c**) OVCAR3 and (**g**) HepG2 cells. Dose–response cytotoxicity of (**d**) OVCAR3, (**i**) OCIAML3, (**j**) K562, (**k**) ARH77 and (**l**) Hela cells. Dose–response cytotoxicity of (**h**) HepG2, (**m**) primary HCC, (**n**) Huh-7, (**o**) SW480 and (**p**) Hela cells. Data are representative of three independent experiments. ****P < 0.0001; ns not significant

To evaluate the antigen-specific cytotoxic potential of TCRm Bi-NbTE in vitro, we performed flow cytometry-based cytotoxicity assays. Expression analysis of public datasets confirmed variable WT1 and GPC3 levels across tumor tissues and tumor lines (Supplementary Fig. S8a-g). Co-culture of T cells with various HLA-A2^+^/GPC3^+^ cells (T2-GPC3_144-152_, HepG2, and primary hepatocellular carcinoma (HCC) cells) or HLA-A2^+^/WT1^+^ cells (T2-WT1_126-134_, OVCAR3, and OCIAML3 cells) at an effector-to-target (E:T) ratio of 10:1 revealed that both TCRm Bi-NbTE induced potent, dose-dependent T cell cytotoxicity (Fig. 3a, d, e, h, i, m). This cytotoxicity was significantly superior to that mediated by the Irrelevant NbTE or T cells alone (Fig. 3b, f). Strikingly, no appreciable lysis was observed against pMHC^−^ cells (T2-irrelevant peptides, T2 cells, K562, ARH77, Huh-7, SW480 or Hela cells) at different concentrations (Fig. 3a, e, j–l, n–p), confirming its tumor cell lysis in a strictly antigen-specific and dose-dependent manner.

Moreover, results demonstrated that TCRm Bi-NbTE-mediated T cell cytotoxicity was dependent on the E:T ratio. TCRm Bi-NbTE induced the maximal lysis of pMHC^+^ cells HepG2 or OVCAR3 at an E:T ratio of 10:1, and reduced but significant lysis at a ratio of 5:1 (Fig. 3c, g). As expected, cytotoxicity occurred only in the presence of T cells, with no lysis observed in their absence, underscoring that the cytotoxicity was entirely T cell-dependent (Supplementary Fig. S9d, e). Besides, TCRm Bi-NbTE was significantly more effective than its anti-TCRm Nb counterpart, highlighting the critical role of CD3 engagement in driving efficient killing. Collectively, these data establish that TCRm Bi-NbTE selectively lyse pMHC^+^ tumors cells by inducing T cell-mediated cytotoxicity in an HLA-A2^+^/WT1^+^ or HLA-A2^+^/GPC3^+^ restricted manner. This precise antigen specificity and potent cytotoxic capacity highlight TCRm Bi-NbTE as a promising therapeutic strategy for targeting intracellular tumor antigens with minimal off-target effects.

### TCRm Bi-NbTE exhibits robust antitumor efficacy in humanized mouse models

**Fig. 4:**
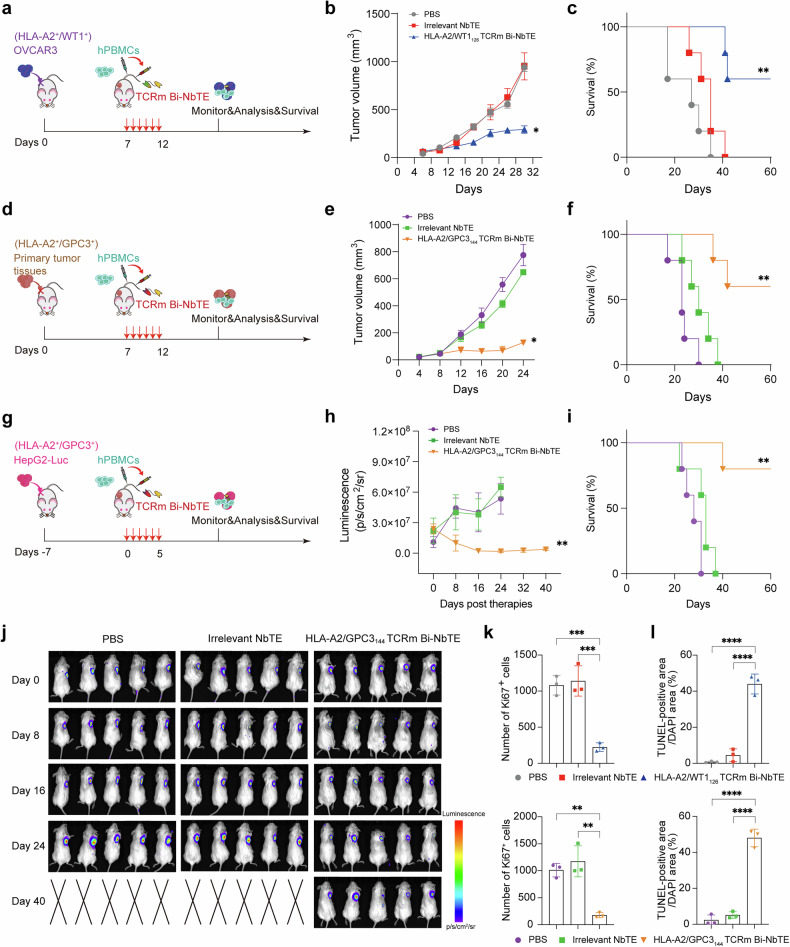
Antitumor efficacy of TCRm Bi-NbTE in multiple xenograft mouse models. Schematic diagram of the experimental timeline. NOD/SCID mice (*n* = 5) were subcutaneously implanted with (**a**) OVCAR3 tumor cells, (**d**) PDX tumors, or (**g**) HepG2-Luc tumor cells, injected intravenously with human PBMCs and were treated daily TCRm Bi-NbTE or control antibodies for six consecutive days. (**b**, **e**) Tumor growth curves. (**c**, **f**, **i**) Kaplan–Meier survival curves. **h** Quantitative analysis of luminescence intensity, and (**j**) representative in vivo bioluminescence imaging of HepG2-Luc tumors. Quantification of (**k**) Ki-67-positive proliferating cells, and (**l**) apoptotic cells in resected tumor tissues from the OVCAR3 or HepG2-Luc model after TCRm Bi-NbTE treatment. *P < 0.05; **P < 0.01; ***P < 0.001; ****P < 0.0001

To evaluate the therapeutic potential of TCRm Bi-NbTE in vivo, we established cell-derived xenograft (CDX) models using HepG2-Luc and OVCAR3 tumor cells, respectively. NOD/SCID mice bearing subcutaneous tumors were randomized into treatment groups and received daily intravenous injections of TCRm Bi-NbTE or control molecules for 6 days following adoptive transfer of human peripheral blood mononuclear cells (PBMCs) (Fig. 4a, g). TCRm Bi-NbTE treatment significantly suppressed tumor growth compared to both control groups, as corroborated by tumor size measurement and in vivo bioluminescent imaging assessment (Fig. 4b, h, j), as well as conferred a significant survival benefit with TCRm Bi-NbTE compared to Irrelevant NbTE or PBS groups (Fig. 4c, i), providing robust evidence for the therapeutic efficacy of the TCRm Bi-NbTE in preclinical models.

To further evaluate its translational potential, we established a patient-derived xenograft (PDX) model using primary human HCC tissue grafted into NOD/SCID mice (Fig. 4d). TCRm Bi-NbTE treatment again significantly inhibited tumor growth and extended survival compared to controls (Fig. 4e, f). Importantly, no significant weight loss (Supplementary Fig. S10a, b) or signs of treatment-related toxicity, as indicated by stable levels of alanine aminotransferase (ALT), aspartate aminotransferase (AST), and pro-inflammatory cytokines including IL-6 or IL-1β (Supplementary Fig. S11a-e). Collectively, these results demonstrate that TCRm Bi-NbTE effectively suppresses tumor progression and improves survival in multiple mouse xenograft models, validating its potent antitumor activity.

**Fig. 5:**
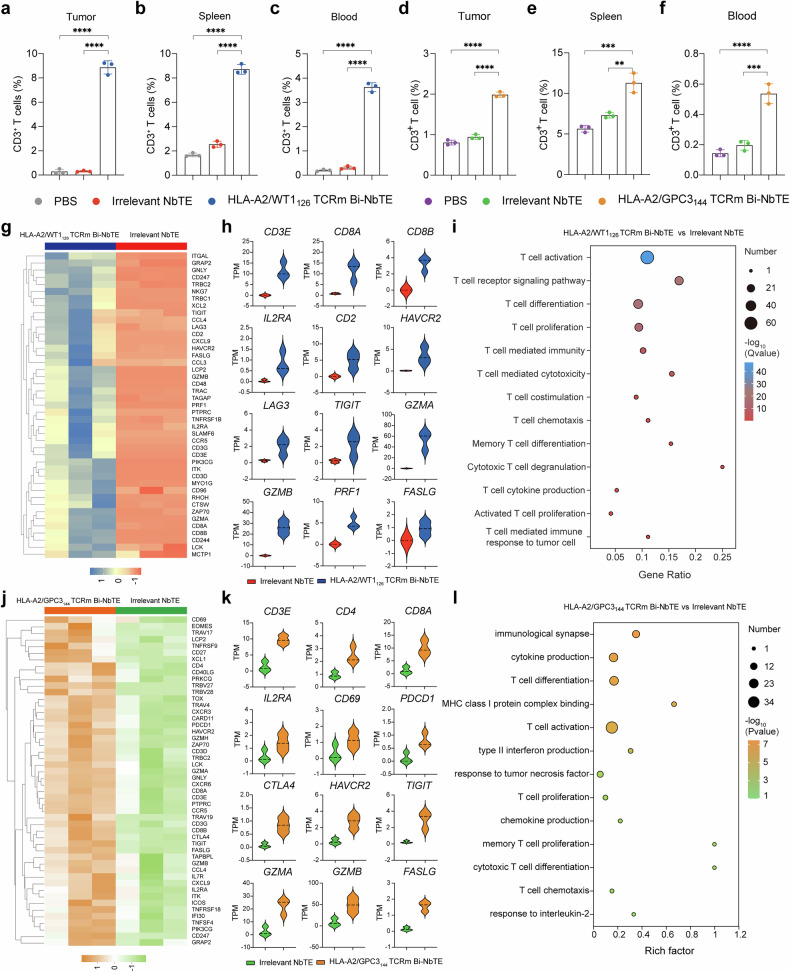
TCRm Bi-NbTE promotes intratumoral T cell functionality by boosting anti-tumor responses. Flow cytometry analysis showing CD3^+^ T cell infiltration in (**a**, **d**) tumor tissues, (**b**, **e**) spleens, and (**c**, **f**) peripheral blood from the OVCAR3 or HepG2-Luc model after TCRm Bi-NbTE treatment. **g**, **j** Heatmaps depicting differential expression of immune-related genes in tumor tissues from TCRm Bi-NbTE-treated versus Irrelevant NbTE-treated mice. **h**, **k** Relative expression of selected key genes associated with T cell activation, proliferation, exhaustion and cytotoxicity in the respective groups. **i**, **l** GO enrichment analysis showing significantly enriched pathways in tumor tissues from TCRm Bi-NbTE-treated versus Irrelevant NbTE-treated mice. **P < 0.01; ***P < 0.001; ****P < 0.0001

We then performed histological analysis of harvested HepG2-Luc or OVCAR3 xenograft tumors. TCRm Bi-NbTE-treated tumors exhibited a significant increase in apoptosis cells and concurrent decrease in Ki-67^+^ proliferating cells compared to Irrelevant NbTE and PBS controls (Fig. 4k, l). Additionally, flow cytometry analysis revealed increased numbers of CD3^+^ T cells within the tumors and spleens of TCRm Bi-NbTE-treated mice (Fig. 5a, b, d, e, Supplementary Fig. S12a-b), with sustained peripheral CD3^+^ T cell counts observed within two weeks post-injection (Fig. 5c, f, Supplementary Fig. S12a-b).

**Fig. 6:**
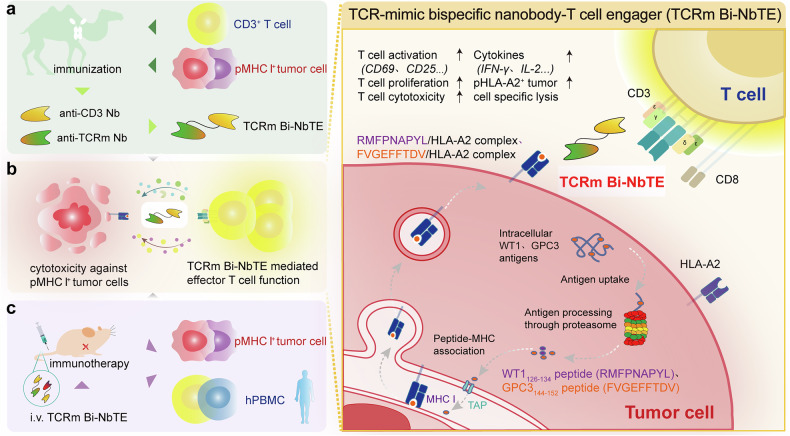
Schematic overview of the TCRm Bi-NbTE platform and its therapeutic mechanism. **a** Workflow outlining the generation of specific anti-TCRm nanobodies and an anti-CD3ε nanobody, and their subsequent engineering into the TCRm Bi-NbTE construct. **b** Proposed working model of the TCRm Bi-NbTE mediated T cell–tumor cell engagement (Left), and therapeutic characterization of TCRm Bi-NbTE molecule targeting the HLA-A2/WT1_126-134_ complexes or HLA-A2/GPC3_144-152_ complexes (Right), redirecting T cell cytotoxicity independent of endogenous TCR specificity. **c** Summary diagram depicting that TCRm Bi-NbTE promotes potent antitumor efficacy in multiple mouse xenograft models, validating this modular platform as a novel strategy for targeting intracellular antigens. Generated by Adobe Illustrator software

Transcriptomic profiling of tumor tissues provided further mechanistic insight. TCRm Bi-NbTE treatment upregulated a broad signature of T cell activity, including lineage markers (*CD3E, CD8A, CD4*), activation markers (*IL2RA, CD69*), cytotoxic effectors (*GzmB, GzmA, PRF1, FASLG*). A moderate increased expression of exhaustion markers (*PDCD1, CTLA4, HAVCR2, LAG3, TIGIT*) when compared to the control groups, consistent with sustained activation signaling accompanying robust effector responses (Fig. 5g, h, j, k). Furthermore, gene ontology (GO) enrichment analysis between the TCRm Bi-NbTE and Irrelevant NbTE groups revealed significant enrichment of pathways related to T cell proliferation, activation, mediated cytotoxicity and immunity, and proinflammatory cytokine signaling (Fig. 5i, l). Taken together, these results demonstrate that TCRm Bi-NbTE mediates robust antitumor efficacy in multiple preclinical models, mediating tumor regression through antigen-specific T cell recruitment, activation, and cytotoxic effector functions (Fig. 6a–c), positioning TCRm Bi-NbTE as a robust and scalable therapeutic platform for expanding T cell-engaging immunotherapy to intracellular tumor targets.

## Discussion

The development of novel TCEs is critical for overcoming the limitations of existing T cell-engaging immunotherapies, particularly for treating malignant tumors driven by intracellular oncoproteins. In this study, we introduced TCRm Bi-NbTE, a first-in-class TCRm nanobody-based bispecific T cell engager that simultaneously targets CD3ε and tumor-associated pMHC complexes (HLA-A2/WT1_126-134_ or HLA-A2/GPC3_144-152_). Our findings demonstrate that TCRm Bi-NbTE potently redirects T cells to induce robust, antigen-restricted activation and cytotoxicity against pMHC⁺ tumors, while sparing pMHC^−^ cells, representing a conceptual advance in TCE design for cancer immunotherapy.

A key distinction between TCRm Bi-NbTE and traditional scFv-based TCEs lies in their structural and biophysical properties of the platforms. Conventional scFv-based constructs often suffer from aggregation-prone architectures and suboptimal tissue penetration. By contrast, TCRm Bi-NbTE utilizes camelid-derived nanobodies, which possess compact size, exceptional stability, and enhanced tumor penetration capability. Moreover, TCRm Bi-NbTE addresses critical challenges that have hindered prior bispecific formats like ImmTAC,^30,32,33^ ESK1-BiTE^23^ or TCR fusion proteins.^34^ By replacing scFvs with nanobodies, TCRm Bi-NbTE circumvents structural instability due to VH-VL domain mismatching and facilitates efficiently production in prokaryotic systems, and ensures effective T cell-tumor cell engagement in tumors even at relatively low pMHC densities. It is noted that ESK1 mediates tumor killing primarily through antibody-dependent cellular cytotoxicity (ADCC), while TCRm Bi-NbTE drives direct, antigen-dependent T cell cytotoxicity, offering a fundamentally distinct mechanism on their respective antitumor potency. Together, our findings underscore the potential of a bispecific VHH-VHH engineering platform, exemplified by the TCRm Bi-NbTE format, to overcome key limitations of native TCRs and existing TCR-T therapies, including low intrinsic affinity and complex heterodimeric structure. Importantly, this format can be readily redirected against other intracellular antigens and neoantigens, thereby establishing a modular and unique platform for unleashing T cell‑mediated antitumor immunity across a wide spectrum of otherwise intractable tumors.

The nanobody scaffold also offers significant potential for clinical applications due to their favorable affinity and scalability for manufacturing.^35^ Leveraging our previously characterized anti-CD3ε Nb, HLA-A2/WT1_126-134_ TCRm Nb and HLA-A2/GPC3_144-152_ TCRm Nb,^19,26^ we successfully produced functional TCRm Bi-NbTE molecules with favorable binding kinetics. Accordingly, TCRm Bi-NbTE exhibited stringent specificity, driving T cell proliferation, activation, cytokine secretion, and cytotoxicity exclusively against antigen-matched targets in vitro, suggesting its promising clinical application as either a monotherapy or in combination with other immunotherapies. Importantly, TCRm Bi-NbTE effectively suppressed tumor suppression and prolonged survival in both PDX and CDX models, accompanied by increased intratumoral T cell infiltration and a transcriptional signature of T cell activation and cytotoxicity. These findings position TCRm Bi-NbTE as a promising candidate with a more streamlined manufacturing process for clinical translation, particularly for cancers with limited treatment options.

Perhaps the most compelling features of TCRm Bi-NbTE are its ability to target intracellular tumor antigens presented on pMHC complexes, therapy expanding the targetable space to include neoantigens and shared intracellular oncoproteins. While direct targeting of surface GPC3 is indeed feasible, our pMHC-centric strategy offers distinct and complementary advantages with potentially superior tumor specificity and avoidance of soluble GPC3 antigen interference. Unlike autologous cell therapies such as CAR-T, which require complex manufacturing and carry risks such as cytokine release syndrome.^36–38^ TCRm Bi-NbTE represents an “off-the-shelf,” pharmacologically tractable modality. Its modular design enables rapid re-targeting to other emerging pMHC complexes. Notably, this strategy is not intended to replace but to complement direct protein-targeting. It represents a strategic choice prioritizing tumor selectivity and serves as a proof-of-concept for a potentially universal platform capable of targeting the vast repertoire of intracellular tumor antigens, with applications across a wide range of tumor types, thereby addressing a major limitation in current TCE immunotherapy.

Despite these promising findings, there are several limitations that need to be addressed in future studies. The current study employed immunodeficient mouse models, which may not fully recapitulate human tumor-immune interactions. Looking forward, TCRm Bi-NbTE can also be evaluated in immunocompetent mouse models expressing humanized CD3ε chains (huCD3ε mice) or HLA-A2 transgenes to reveal the key factors governing clinical responses.^39^ Although no serious adverse effects related to the treatment were observed in mouse models, comprehensive evaluation of on-target/off-tumor toxicity and immunogenicity will require future studies in humanized immune models or non-human primates. Additionally, while transcriptomic data revealed upregulated T cell effector pathways, the upstream regulators driving these effects require further mechanistic elucidation. Structural studies are needed to delineate the precise molecular interactions governing specificity. Importantly, to improve patient convenience and treatment compliance by reducing dosing frequency, we are developing a half-life extended format by fusing an anti-human serum albumin (HSA) nanobody, which is expected to significantly improve the pharmacokinetic properties and facilitate subsequent clinical applicability.^40,41^ Although Nbs generally exhibit low immunogenicity, humanization strategies will be essential for clinical translation.^42,43^ Finally, exploring combination strategies with CAR-T cell therapy, cancer vaccines, or immune checkpoint inhibitors could further synergize its therapeutic efficacy in cancer immunotherapy.

In conclusion, by synergizing the precision of nanobody-based T cell engager with the biophysical advantages of TCRm nanobodies, the TCRm Bi-NbTE overcomes the structural and functional limitations of conventional TCEs. It enables the specific redirection of T cells against intracellular tumor antigens with high potency. This study not only validates a novel therapeutic strategy but also provides a versatile and scalable engineering platform targeting intracellular oncoproteins, setting the stage for broader clinical applications in cancer immunotherapy.

## Methods and materials

### Cell lines and animal experiments

PBMCs were isolated from HLA-typed healthy donors via Ficoll density gradient centrifugation (Solarbio, China), and cultured in complete RPMI-1640 medium containing 100 IU/mL recombinant human IL-2 (PeproTech, UK). Written informed consent was obtained from all donors under protocols approved by the Ethics Committee of Guangxi Medical University (No. GXMU-IRB-APR-2026-018-P). HepG2, Huh-7, SW480, and Hela cells were maintained in DMEM medium (Gibco, USA) supplemented with 10% fetal bovine serum (FBS) and 1% penicillin–streptomycin at 37 °C under 5% CO_2_. OVCAR3, OCIAML3, K562, ARH77 and TAP-deficient T2 cells were cultured in RPMI-1640 medium (Gibco, USA) with identical supplements and conditions.

Female NOD/SCID mice (6–8 weeks old) were purchased from Vital River Laboratories (Beijing, China) and housed under specific pathogen-free (SPF) conditions. All experimental protocols with animals were approved by the Institutional Animal Care and Use Committee (IACUC) of Guangxi Medical University (No. 202111026) and conducted in accordance with ARRIVE guidelines.

### Peptides

HLA-A*02:01-restricted peptides *FVGEFFTDV* (GPC3_144-152_), *RMFPNAPYL* (WT1_126-134_), *FLGEAFDGA* (SAA1), *FLDEFVTFL* (PLCG1), *FMDEFFEQV* (STX1A), *FMFEYFSPV* (GRIN2C), *RLFPLAWTV* (EFNA1), *QLLPNLAEL* (FBXL16), *KLPPNVVAV* (GPD1), *RLFPIAWRL* (CYP3A4), along with alanine-substituted peptide variants, were synthesized by GenScript (>95% purity, HPLC-verified). Peptides were dissolved in dimethyl sulfoxide (DMSO) at 50 mg/mL, further diluted in serum-free RPMI-1640 at 5 mg/mL working stock, aliquoted, and stored at −80 °C.

### Generation and validation of TCRm Bi-NbTE

TCRm Bi-NbTE is a bispecific nanobody comprised of anti-human HLA-A2/GPC3_144-152_ TCRm nanobody or anti-human HLA-A2/WT1_126-134_ TCRm nanobody at the N-terminus and an anti-human CD3ε nanobody at the C-terminus. DNA fragments encoding the TCRm Bi-NbTE were synthesized by GenScript and subcloned into *p*ET-30a expression vector using genetic engineering technology. A His tag and a Flag tag were inserted to the C-terminnus for purification and detection.

The recombinant plasmid was transformed into *E. coli* BL21 (DE3) competent cells for heterologous expression. Following induction, bacterial cultures were harvested by centrifugation (12,000 rpm, 30 min), and pellets were resuspended in PBS, and lysed by ultrasonication. Inclusion bodies were collected through sequential PBS washing, solubilized in 8 M urea buffer containing 20–250 mM imidazole using a stepwise elution gradient, and purified by Ni^2+^‒NTA resin (GenScript, China) according to the manufacturer’s protocol. Subsequently, proteins were dialyzed against refolding buffer containing 50 mM Tris-HCl, 150 mM NaCl, 2 mM DTT (pH 8.0), and sterilized through a 0.22 μm filter. The purity proteins were assessed by SEC‒HPLC analysis and SDS‒PAGE under reducing conditions and visualized with Coomassie brilliant blue staining. Binding kinetics and affinity for target antigens were determined by BLI using an Octet R8 system (ForteBio, USA).

### Western blotting analysis

TCRm Bi-NbTE expression was detected by Western blotting using established protocols.^18^ Briefly, purified recombinant proteins were separated on SDS‒PAGE under reducing conditions and electrophoretically transferred onto 0.45 μm-polyvinylidene fluoride membranes using a tank blotting system (Bio-Rad, USA) in ice-cold transfer buffer. After incubation with 5% (w/v) skim milk blocking solution, membranes were incubated overnight at 4 °C with horseradish peroxidase (HRP)-conjugated anti-His-tag monoclonal antibody (1:3000 dilution, Abcam). The immunoreactive bands were visualized using BeyoECL Plus chemiluminescent substrate (Beyotime, China) and analyzed using a ChemiDoc MP Imaging System coupled with Image Lab software (Bio-Rad, USA).

### Enzyme‑linked immunosorbent assay

The simultaneous antigen-binding capability of TCRm Bi-NbTE was evaluated by a two-step sandwich ELISA. Briefly, hCD3 epsilon (ACRO, CDEH5256) was immobilized as the capture reagents. After blocking, plates were incubated with serial dilutions of TCRm Bi-NbTE, followed by addition of biotinylated HLA-A2/WT1_126-134_ complex protein or HLA-A2/GPC3_144-152_ complex protein (ACRO, HLW-H82E5, HLG-H82E4) as the detection reagents. Bound complexes were subsequently detected using streptavidin-HRP and 3,3’,5,5’-tetramethylbenzidine substrate. The reaction was terminated with 50 μL of 1 M H_2_SO_4_, and absorbance was immediately measured at 450 nm using a microplate reader (Tecan, Mannedorf, Switzerland). In addition, the levels of IL-2 and IFN-γ were quantified using commercial ELISA kits (Liankebio, China) according to the manufacturer’s protocol. Absorbance at 450 nm was converted to concentration values via standard curves.

### Enzyme-linked immunospot assay

The frequency of IFN-γ-secreting cells was quantified using a human IFN-γ precoated ELISPOT Kit (Dakewe, China) according to the manufacturer’s instructions. Briefly, T cells and target cells were co-cultured in the presence of TCRm Bi-NbTE or controls for 24 h. After washing, plates were incubated with biotinylated IFN-γ antibody solutions for 1 h at 37 °C, followed by streptavidin HRP-conjugated solutions for 1 h at 37 °C. Subsequently, spots were developed using 3-amino-9-ethylcarbazole substrate for 30 min. The reaction was terminated with ddH_2_O and images were acquired and quantified using an ImmunoSpot analyzer (Cellular Technology Limited, USA).

### T cell proliferation assay

T cell proliferation was assessed using the CellTrace™ 5,6-carboxyfluorescein diacetate succinimidyl ester (CFSE) cell proliferation kit (Thermo Fisher Scientific, USA) according to the manufacturer’s protocol. Briefly, T cells were labeled with 5 μM CFSE in PBS for 10 min at 4 °C, and then quenched with ice-cold complete RPMI-1640 medium, followed by three washes. CFSE-labeled T cells were co-cultured with mitomycin C-treated tumor cells at an E: T ratio of 10:1 in the presence of TCRm Bi-NbTE or control molecules for an additional 3 days. Cells were harvested and analyzed by flow cytometry (BD FACSCanto II, BD Biosciences). Proliferation was quantified based on CFSE dilution using FlowJo v10.9.1 software.

### T cell activation and memory phenotype assay

For activation marker analysis, T cells were co-cultured with target cell lines at E:T ratio of 10:1 in the presence of TCRm Bi-NbTE or control molecules. After 24 h of incubation at 37 °C, 5% CO_2_, cells were harvested and stained with PE-anti-CD4, PE-anti-CD8, APC-anti-CD69, APC-anti-CD25 and APC-anti-CD107a (BioLegend, USA). For memory subset analysis, co-cultures were maintained for 14 days with periodic medium replenishment. Cells were then stained with PE-Cy7-anti-CD45RA and APC-anti-CCR7 (BioLegend, USA). Samples were acquired on a BD FACSCanto II system (BD Biosciences) and data were analyzed with FlowJo v10.9.1 software.

### In vitro cytotoxicity assay

The specific cytotoxicity of TCRm Bi-NbTE was measured using a flow cytometry-based killing assay. Target cells were labeled with 5 μM CFSE according to the manufacturer’s protocols. T cells were then co-cultured with labeled target cells at E: T ratios of 1:1, 5:1, and 10:1 in the presence of titrated concentrations of TCRm Bi-NbTE or control molecules. After 16 h of incubation at 37 °C, 5% CO_2_, cells were stained with 1 μg/mL 7-Aminoactinomycin D (7-AAD; AAT Bioquest) according to the manufacturer’s protocol. The percentage of CFSE⁺7-AAD⁺ cells within the target cell gate was determined by flow cytometry. Specific lysis was calculated as: (% sample lysis− % spontaneous lysis)/(% maximum lysis − % spontaneous lysis) × 100.

### In vivo efficacy study

To establish CDX models, HepG2-Luc cells (1 × 10^6^ cells/mouse) or OVCAR3 cells (1 × 10^6^ cells/mouse) were subcutaneously implanted into the right axilla of NOD/SCID mice. Seven days post-implantation, mice were randomized into treatment groups (*n* = 5) based on initial tumor volume, PBMCs (1 × 10^7^ cells/mouse) were administered via tail vein injection. Mice received daily intravenous injections of TCRm Bi-NbTE (20 μg/mouse) or control molecules for six consecutive days. Control groups received equal volumes of PBS. Tumor burden was monitored weekly by bioluminescence imaging using in vivo animal imaging system AniView (BLT, China). Tumor dimensions were measured every 4 days using digital calipers, and volumes were calculated as (length × width^2^)/2. The survival of mice was also be evaluated, body weight was recorded simultaneously to assess treatment-related toxicity. Mice were humanely euthanized when tumor length exceeded 15 mm, exceeded 20% body weight loss, or ulceration. For endpoint analyses, serum IL-6 and IL-1β levels were measured using ELISA kits (Liankebio, China) according to the manufacturers’ instructions. ALT and AST levels were measured using an automatic biochemical analyzer (Catalyst One, IDEXX, USA). Tumors, spleens, and blood were harvested for further ex vivo analysis.

### Patient-derived xenograft model

PDX models were established as previously described.^18^ In brief, primary human HCC tissue fragments from surgical resections, obtained and written informed consent was obtained from all donors under protocols approved by the Ethics Committee of Guangxi Medical University (No. GXMU-IRB-APR-2026-018-P), were mechanically dissociated ~2 mm^3^ in ice-cold saline solution. Fragments were mixed with Matrigel matrix (Corning, USA; 1:1 v/v) and subcutaneously implanted into the lower dorsal flank of NOD/SCID mice. Seven days post-engraftment, mice were randomized (n = 5) and treated following the same PBMC and TCRm Bi-NbTE administration regimen as described for the CDX models. Tumor growth and survival period of mice were monitored.

### RNA sequencing assay

RNA sequencing was performed by Gene Denovo Biotechnology Co., Ltd. (Guangzhou, China) or Majorbio Bio-pharm Technology Co., Ltd. (Shanghai, China). Raw sequencing reads were quality-filtered via Trimmomatic (quality >20), and then aligned to the human reference genome (GRCh38) (STAR v2.7.10a), and transcript abundance was quantified as transcripts per million (Salmon v1.6.0). Differentially expressed genes (DEGs) were identified using DESeq2, and the results were visualized as heatmaps. Immune-related gene signatures were characterized by enrichment analyses based on curated public gene sets.

### Histological studies

For Ki-67 staining, formalin-fixed, paraffin-embedded tumor sections (5 μm) underwent antigen retrieval, endogenous peroxidase quenching and blocking. Primary anti-Ki-67 antibody (MXB Biotechnologies, China) was applied overnight at 4 °C. and then incubated with the corresponding secondary antibody according to standard protocols. Signal amplification was performed using diaminobenzidine (DAB) substrate, and nuclei were counterstained with hematoxylin. Apoptotic cells in tumor tissues were detected using a one-step TUNEL apoptosis detection kit (Beyotime, China) following the manufacturer’s protocol. Briefly, sections were deparaffinized, rehydrated, and treated with proteinase K (20 μg/mL), and then incubated with TdT reaction mix. Nuclei were counterstained with DAPI (1 μg/mL). Images were captured using a Nikon microscope and analyzed NIS-Elements software (Nikon, Japan).

### Statistical analysis

Statistical analysis and plotting were performed using GraphPad Prism 9 software (GraphPad Software, San Diego, CA). Data are presented as mean ± standard deviation (SD) from three independent experiments. Significance was determined by one-way analysis of variance (ANOVA) with Tukey’s multiple comparison or Student’s *t* test. *P* < 0.05 was considered statistically significant (*P < 0.05; **P < 0.01; ***P < 0.001; ****P < 0.0001).

## Supplementary information

Supplementary Materials

## Data Availability

The authors confirm that the data supporting the findings of this study are available within the article and its supplementary materials. The raw sequence data reported in this paper have been deposited in the Genome Sequence Archive in National Genomics Data Center, China National Center for Bioinformation/Beijing Institute of Genomics, Chinese Academy of Sciences (GSA: CRA040975) which are accessible at https://ngdc.cncb.ac.cn/gsa.

## References

[1] Bargou, R. C. The expanding success of T cell-engaging bispecific antibodies. *Nat. Cancer* **4**, 1054–1055 (2023). 37316727 10.1038/s43018-023-00586-z

[2] Dagher, O. K., Schwab, R. D., Brookens, S. K. & Posey, A. D. Jr Advances in cancer immunotherapies. *Cell* **186**, 1814–1814.e1811 (2023). 37059073 10.1016/j.cell.2023.02.039

[3] Fenis, A., Demaria, O., Gauthier, L., Vivier, E. & Narni-Mancinelli, E. New immune cell engagers for cancer immunotherapy. *Nat. Rev. Immunol.* **24**, 471–486 (2024). 38273127 10.1038/s41577-023-00982-7

[4] van de Donk, N. & Zweegman, S. T-cell-engaging bispecific antibodies in cancer. *Lancet* **402**, 142–158 (2023). 37271153 10.1016/S0140-6736(23)00521-4

[5] Goebeler, M. E. & Bargou, R. C. T cell-engaging therapies—BiTEs and beyond. *Nat. Rev. Clin. Oncol.* **17**, 418–434 (2020). 32242094 10.1038/s41571-020-0347-5

[6] Gupta, S. et al. Blinatumomab in Standard-Risk B-cell acute lymphoblastic leukemia in children. *N. Engl. J. Med* **392**, 875–891 (2025). 39651791 10.1056/NEJMoa2411680 PMC11864901

[7] Litzow, M. R. et al. Blinatumomab for MRD-negative acute lymphoblastic leukemia in adults. *N. Engl. J. Med.* **391**, 320–333 (2024). 39047240 10.1056/NEJMoa2312948 PMC11334054

[8] Hodder, A. et al. Blinatumomab for first-line treatment of children and young persons with B-ALL. *J. Clin. Oncol.* **42**, 907–914 (2024). 37967307 10.1200/JCO.23.01392

[9] Boucher, L. E. et al. “Stapling” scFv for multispecific biotherapeutics of superior properties. *MAbs* **15**, 2195517 (2023). 37074212 10.1080/19420862.2023.2195517 PMC10120459

[10] Lou, H. & Cao, X. Antibody variable region engineering for improving cancer immunotherapy. *Cancer Commun.* **42**, 804–827 (2022). 10.1002/cac2.12330 PMC9456695 35822503

[11] Chandran, S. S. & Klebanoff, C. A. T cell receptor-based cancer immunotherapy: Emerging efficacy and pathways of resistance. *Immunol. Rev.* **290**, 127–147 (2019). 31355495 10.1111/imr.12772 PMC7027847

[12] Klebanoff, C. A., Chandran, S. S., Baker, B. M., Quezada, S. A. & Ribas, A. T cell receptor therapeutics: immunological targeting of the intracellular cancer proteome. *Nat. Rev. Drug Discov.* **22**, 996–1017 (2023). 37891435 10.1038/s41573-023-00809-z PMC10947610

[13] Cheever, M. A. et al. The prioritization of cancer antigens: a National Cancer Institute pilot project to accelerate translational research. *Clin. Cancer Res.* **15**, 5323–5337 (2009). 19723653 10.1158/1078-0432.CCR-09-0737 PMC5779623

[14] Hamers-Casterman, C. et al. Naturally occurring antibodies devoid of light chains. *Nature* **363**, 446–448 (1993). 8502296 10.1038/363446a0

[15] Sun, S. et al. Nanobody: a small antibody with big implications for tumor therapeutic strategy. *Int. J. Nanomed.* **16**, 2337–2356 (2021). 10.2147/IJN.S297631 PMC7997558 33790553

[16] Yong Joon Kim, J., Sang, Z., Xiang, Y., Shen, Z. & Shi, Y. Nanobodies: robust miniprotein binders in biomedicine. *Adv. Drug Deliv. Rev.* **195**, 114726 (2023). 36754285 10.1016/j.addr.2023.114726 PMC11725230

[17] Asaadi, Y., Jouneghani, F. F., Janani, S. & Rahbarizadeh, F. A comprehensive comparison between camelid nanobodies and single chain variable fragments. *Biomark. Res.* **9**, 87 (2021). 34863296 10.1186/s40364-021-00332-6 PMC8642758

[18] Ding, Z. et al. Nanobody-based trispecific T cell engager (Nb-TriTE) enhances therapeutic efficacy by overcoming tumor-mediated immunosuppression. *J. Hematol. Oncol.* **16**, 115 (2023). 38031188 10.1186/s13045-023-01507-4 PMC10688028

[19] Yang, X. M. et al. Nanobody-based bispecific T-cell engager (Nb-BiTE): a new platform for enhanced T-cell immunotherapy. *Signal Transduct. Target Ther.* **8**, 328 (2023). 37661200 10.1038/s41392-023-01523-3 PMC10475457

[20] Pishesha, N., Harmand, T. J. & Ploegh, H. L. A guide to antigen processing and presentation. *Nat. Rev. Immunol.* **22**, 751–764 (2022). 35418563 10.1038/s41577-022-00707-2

[21] Salzler, R. et al. CAR T cells based on fully human T cell receptor-mimetic antibodies exhibit potent antitumor activity in vivo. *Sci. Transl. Med.* **17**, eado9371 (2025). 40138458 10.1126/scitranslmed.ado9371

[22] Augsberger, C. et al. Targeting intracellular WT1 in AML with a novel RMF-peptide-MHC-specific T-cell bispecific antibody. *Blood* **138**, 2655–2669 (2021). 34280257 10.1182/blood.2020010477 PMC9037755

[23] Dao, T. et al. Therapeutic bispecific T-cell engager antibody targeting the intracellular oncoprotein WT1. *Nat. Biotechnol.* **33**, 1079–1086 (2015). 26389576 10.1038/nbt.3349 PMC4600043

[24] Minagawa, A. et al. Enhancing T cell receptor stability in rejuvenated iPSC-derived T cells improves their use in cancer immunotherapy. *Cell Stem Cell* **23**, 850–858.e854 (2018). 30449714 10.1016/j.stem.2018.10.005

[25] Shimizu, Y. et al. Cancer immunotherapy-targeted glypican-3 or neoantigens. *Cancer Sci.* **109**, 531–541 (2018). 29285841 10.1111/cas.13485 PMC5834776

[26] Li, H. et al. Nanobody-based CAR T cells targeting intracellular tumor antigens. *Biomed. Pharmacother.* **156**, 113919 (2022). 36411612 10.1016/j.biopha.2022.113919

[27] Haus-Cohen, M. & Reiter, Y. Harnessing antibody-mediated recognition of the intracellular proteome with T cell receptor-like specificity. *Front. Immunol.* **15**, 1486721 (2024). 39650646 10.3389/fimmu.2024.1486721 PMC11621052

[28] Yang, X. et al. Facile repurposing of peptide-MHC-restricted antibodies for cancer immunotherapy. *Nat. Biotechnol.* **41**, 932–943 (2023). 36593402 10.1038/s41587-022-01567-w PMC10344781

[29] Dao, T. et al. A TCR mimic monoclonal antibody reactive with the “public” phospho-neoantigen pIRS2/HLA-A*02:01 complex. *JCI Insight* **7**, e151624 (2022). 35260532 10.1172/jci.insight.151624 PMC8983142

[30] Dhillon, S. Tebentafusp: first approval. *Drugs* **82**, 703–710 (2022). 35364798 10.1007/s40265-022-01704-4

[31] Yarmarkovich, M. et al. Targeting of intracellular oncoproteins with peptide-centric CARs. *Nature* **623**, 820–827 (2023). 37938771 10.1038/s41586-023-06706-0 PMC10665195

[32] Guc, E. et al. Tebentafusp, a T cell engager, promotes macrophage reprogramming and in combination with IL-2 overcomes macrophage immunosuppression in cancer. *Nat. Commun.* **16**, 2374 (2025). 40064880 10.1038/s41467-025-57470-w PMC11893752

[33] Lopez, J. S. et al. Phase 1 study of IMCnyeso, a T cell receptor bispecific ImmTAC targeting NY-ESO-1-expressing malignancies. *Cell Rep. Med.* **6**, 101994 (2025). 40054461 10.1016/j.xcrm.2025.101994 PMC12047507

[34] Householder, K. D. et al. De novo design and structure of a peptide-centric TCR mimic binding module. *Science* **389**, 375–379 (2025). 40705894 10.1126/science.adv3813 PMC12313176

[35] Alexander, E. & Leong, K. W. Discovery of nanobodies: a comprehensive review of their applications and potential over the past five years. *J. Nanobiotechnol.* **22**, 661 (2024). 10.1186/s12951-024-02900-y PMC11515141 39455963

[36] Sterner, R. C. & Sterner, R. M. CAR-T cell therapy: current limitations and potential strategies. *Blood Cancer J.* **11**, 69 (2021). 33824268 10.1038/s41408-021-00459-7 PMC8024391

[37] Liu, B. et al. Design of high-specificity binders for peptide-MHC-I complexes. *Science* **389**, 386–391 (2025). 40705892 10.1126/science.adv0185 PMC13077772

[38] Johansen, K. H. et al. De novo-designed pMHC binders facilitate T cell-mediated cytotoxicity toward cancer cells. *Science* **389**, 380–385 (2025). 40705893 10.1126/science.adv0422

[39] Belmontes, B. et al. Immunotherapy combinations overcome resistance to bispecific T cell engager treatment in T cell-cold solid tumors. *Sci. Transl. Med.* **13**, eabd1524 (2021). 34433637 10.1126/scitranslmed.abd1524

[40] Keam, S. J. Ozoralizumab: first approval. *Drugs* **83**, 87–92 (2023). 36509938 10.1007/s40265-022-01821-0

[41] Meetze, K., Mehta, N. K., Li, B., Michaelson, J. S. & Baeuerle, P. A. CLN-978, a novel half-life extended CD19/CD3/HSA-specific T cell-engaging antibody construct with potent activity against B-cell malignancies with low CD19 expression. *J. Immunother. Cancer* **11**, e007398 (2023). 37586770 10.1136/jitc-2023-007398 PMC10432633

[42] Gordon, G. L., Raybould, M. I. J., Wong, A. & Deane, C. M. Prospects for the computational humanization of antibodies and nanobodies. *Front. Immunol.* **15**, 1399438 (2024). 38812514 10.3389/fimmu.2024.1399438 PMC11133524

[43] Rossotti, M. A., Belanger, K., Henry, K. A. & Tanha, J. Immunogenicity and humanization of single-domain antibodies. *FEBS J.* **289**, 4304–4327 (2022). 33751827 10.1111/febs.15809

