# Supplementary material for: TCR-mimic bispecific nanobody-based T cell engager targeting intracellular tumor antigens for cancer immunotherapy
Source: Signal Transduct Target Ther. 2026 Jun 25;11:247. doi: 10.1038/s41392-026-02745-x (PMC13294373; doi:10.1038/s41392-026-02745-x)
Supplement: Supplementary file 1 — Supplementary Materials [file 41392_2026_2745_MOESM1_ESM.docx]

Supplementary Materials for

TCR-mimic bispecific nanobody-based T cell engager targeting intracellular tumor antigens for cancer immunotherapy

Ziqiang Ding^1, 2#^, Shuyang Sun^1#^, Xiaomei Yang^1^, Xianing Huang^1^, Xiaoqiong Hou^1^, ShenXia Xie^1^, Aiqun Liu^1^, Xiaoling Lu^1^*

^1^ School of Basic Medical Sciences/ College of Stomatology/ Hospital of Stomatology/ Guangxi Key Laboratory of Nanobody Research/ Guangxi Nanobody Engineering Research Center, Guangxi Medical University, Nanning 530021, China;

^2^ Department of Laboratory Medicine, Nanjing Drum Tower Hospital, Affiliated Hospital of Medical School, Nanjing University, Nanjing 210008, China;

^#^ These authors contributed equally to this work.

* Correspondence to Xiaoling Lu (luxiaoling@gxmu.edu.cn).

**This PDF file includes:**

Figures. S1 to S12


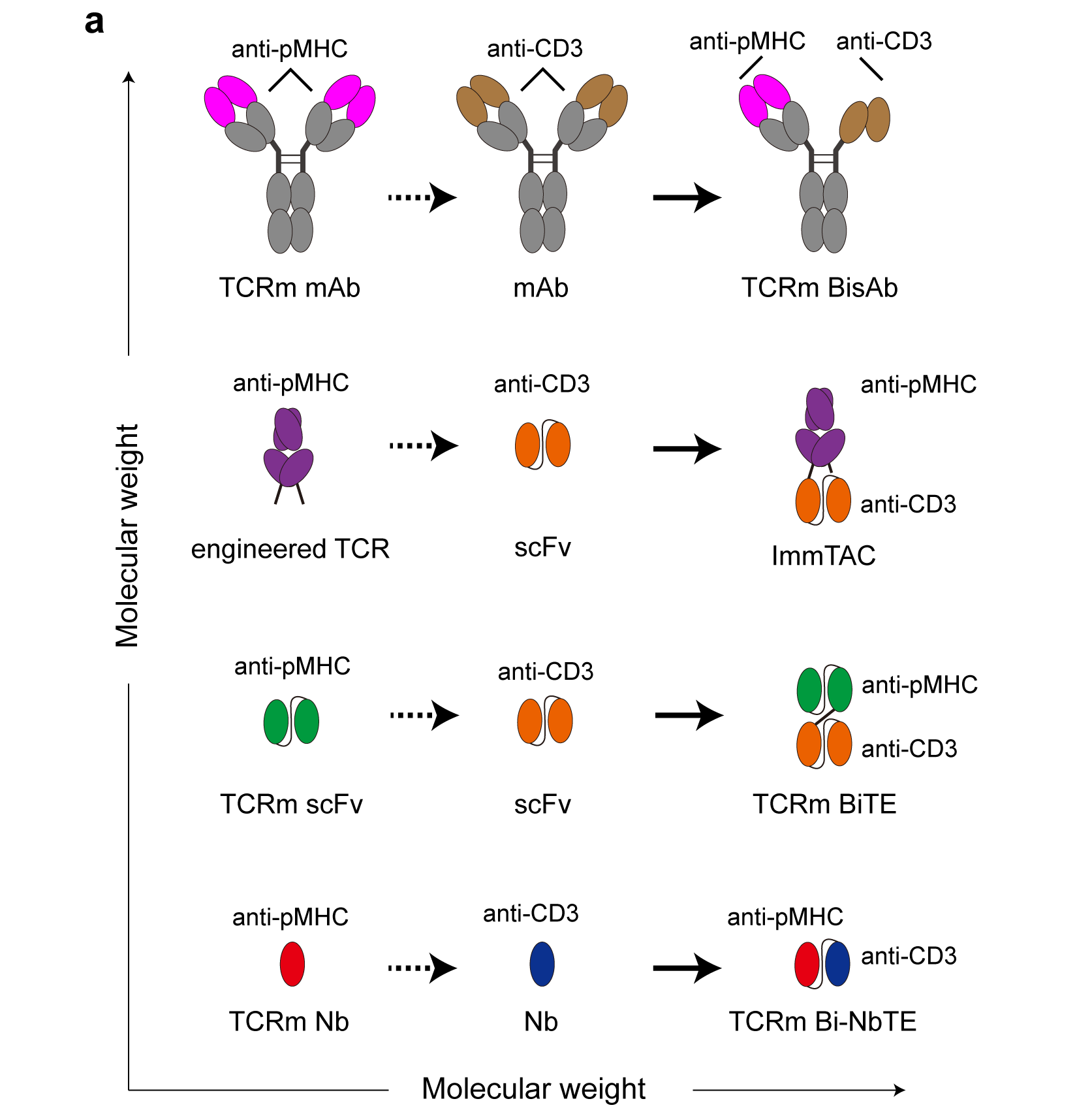


Figure. S1.

**Architectural overview of TCRm antibodies and their derivates.** (a) Schematic illustration of conventional TCR-mimic monoclonal antibodies (TCRm mAb), engineered TCR, TCRm scFv and TCRm Nb. Four representative formats incorporating anti-CD3 engagement modules are shown for comparison. Abbreviations: BisAb: bispecific monoclonal antibody, ImmTAC: immune-mobilising monoclonal TCRs against cancer, BiTE: bispecific T-cell engager, Bi-NbTE: bispecific nanobody-based T-cell engager.


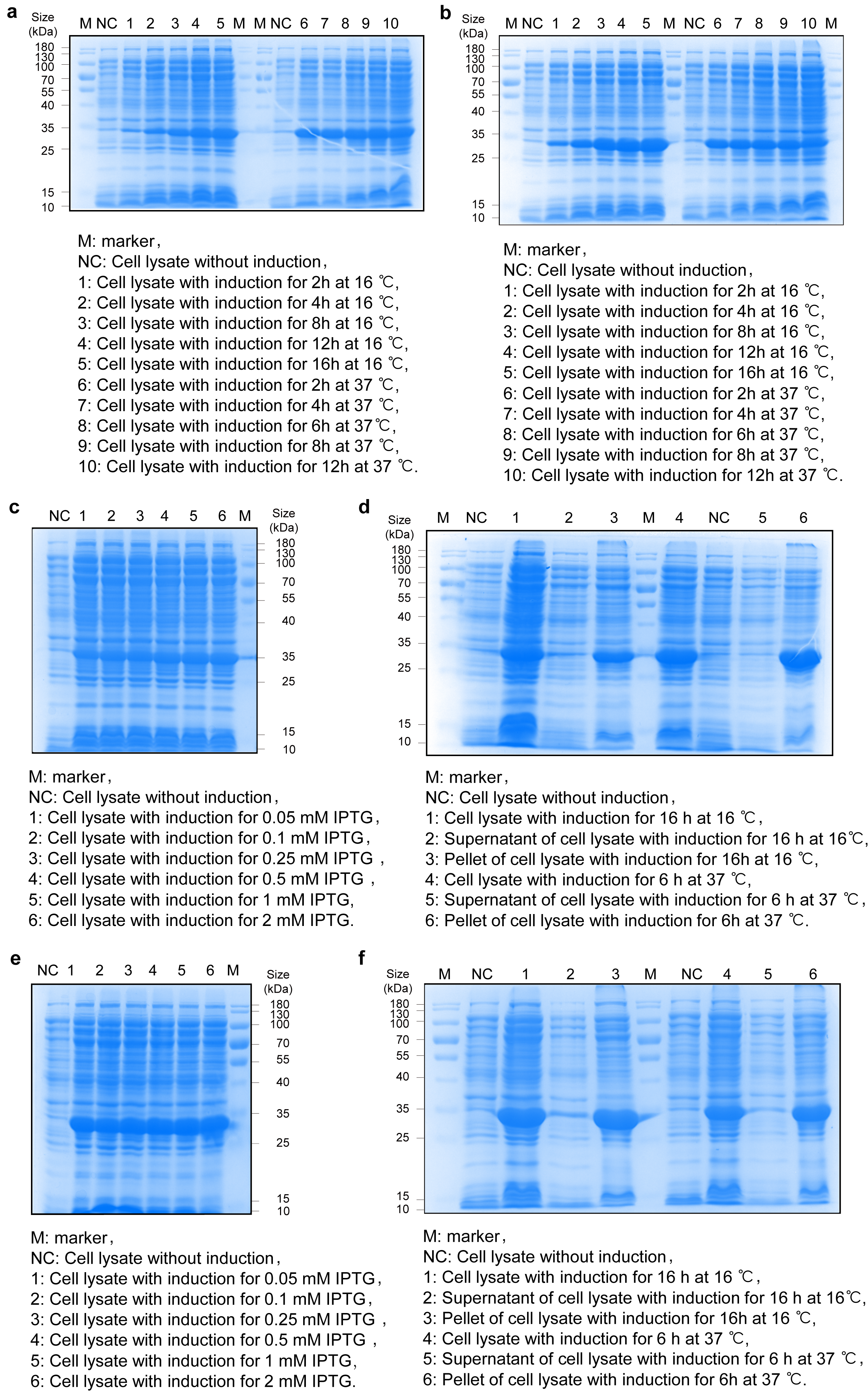


Figure. S2.

**Optimization of induction conditions for TCRm Bi-NbTE protein expression.** (a, c, d) Expression profiles of HLA-A2/WT1_126_ TCRm Bi-NbTE protein under various induction times, temperatures and IPTG concentrations. SDS–PAGE analysis showed that the optimal induction at 0.5 mM IPTG, 37 °C for 6 h. (b, e, f) Expression profiles of HLA-A2/GPC3_144_ TCRm Bi-NbTE protein under various induction times, temperatures and IPTG concentrations. SDS–PAGE analysis showed that the optimal induction at 0.5 mM IPTG, 16 °C for 16 h.


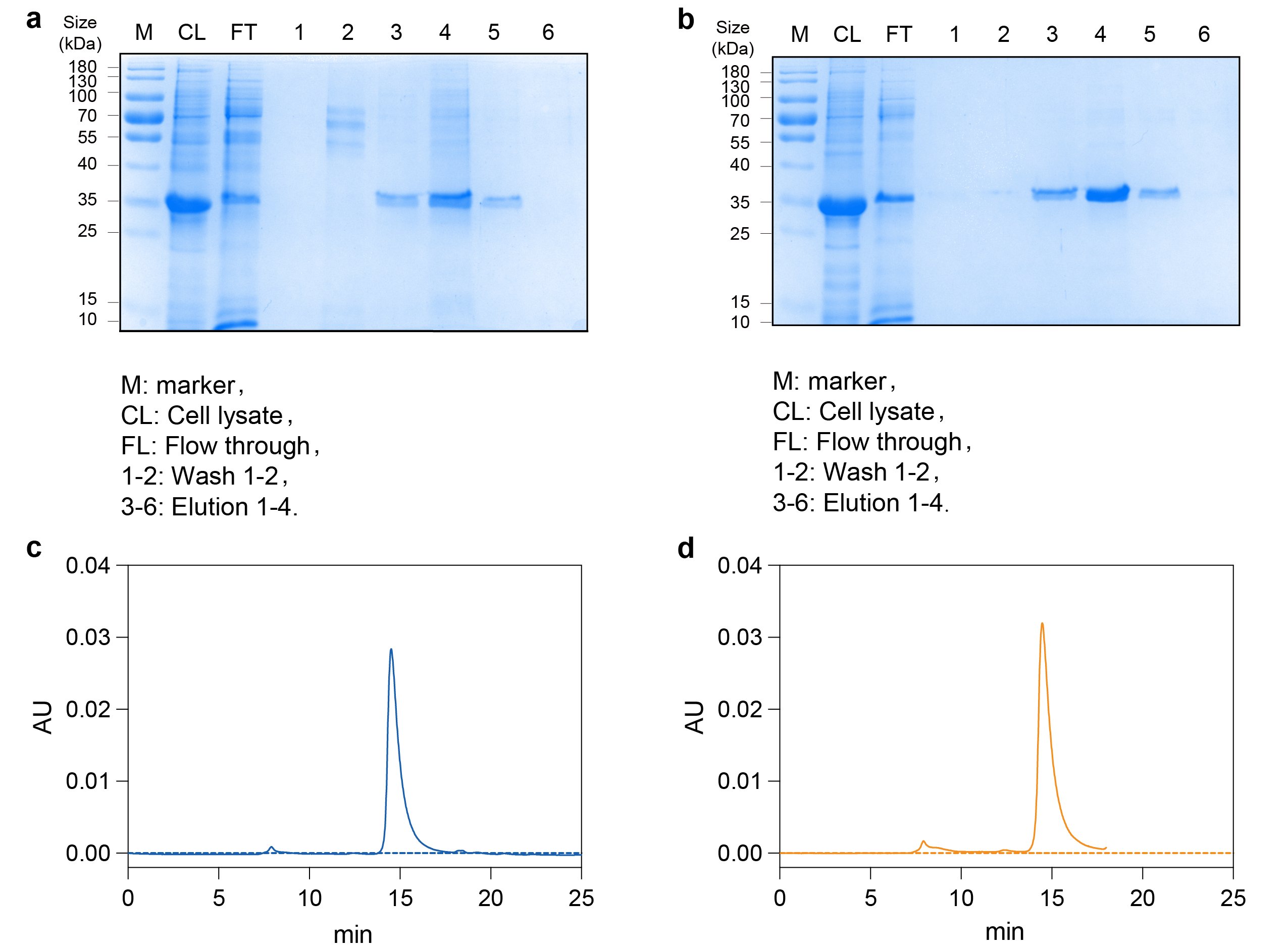


Figure. S3.

**Purification of TCRm Bi-NbTE proteins.** (a, b) SDS–PAGE analysis of elution fractions from Ni^2+^–NTA resin purification of (a) HLA-A2/WT1_126_ TCRm Bi-NbTE and (b) HLA-A2/GPC3_144_ TCRm Bi-NbTE using a stepwise imidazole gradient. (c, d) SEC–HPLC profiles of the final purified (c) HLA-A2/WT1_126_ TCRm Bi-NbTE and (d) HLA-A2/GPC3_144_ TCRm Bi-NbTE respectively, confirming high monomeric purity.


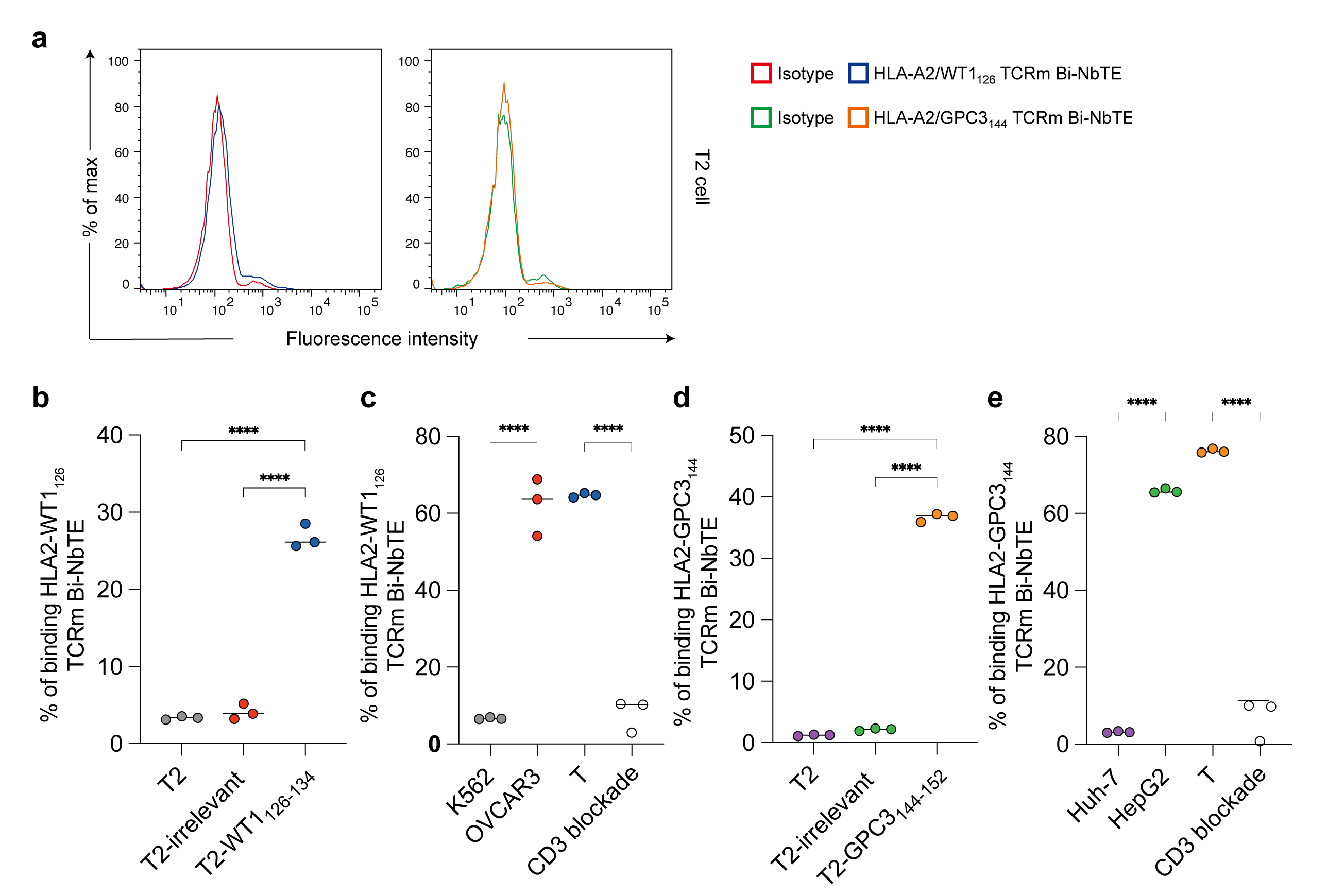


Figure. S4.

**Specific binding capacity of TCRm Bi-NbTE determined by flow cytometry.** (a) Binding analysis of HLA-A2/WT1_126_ TCRm Bi-NbTE or HLA-A2/GPC3_144_ TCRm Bi-NbTE to unpulsed T2 controls by flow cytometry. (b, c) Quantitative bar graphs showing the binding of HLA-A2/WT1_126_ TCRm Bi-NbTE to T2 cells pulsed with the cognate WT1_126-134_ peptide, irrelevant peptides, or unpulsed, as well as to K562 cells (HLA-A2⁻/WT1⁺), OVCAR3 cells (HLA-A2⁺/WT1⁺), primary human T cells with or without pre-blocking by soluble recombinant CD3ε protein. (d, e) Quantitative bar graphs showing the binding of HLA-A2/GPC3_144_ TCRm Bi-NbTE to T2 cells pulsed with the cognate GPC3_144-152_ peptide, irrelevant peptides, or unpulsed, as well as to Huh-7 cells (HLA-A2⁻/GPC3⁺), HepG2 cells (HLA-A2⁺/GPC3⁺), primary human T cells, with or without pre-blocking by soluble recombinant CD3ε protein. Data are representative of three independent experiments. *****P* < 0.0001.


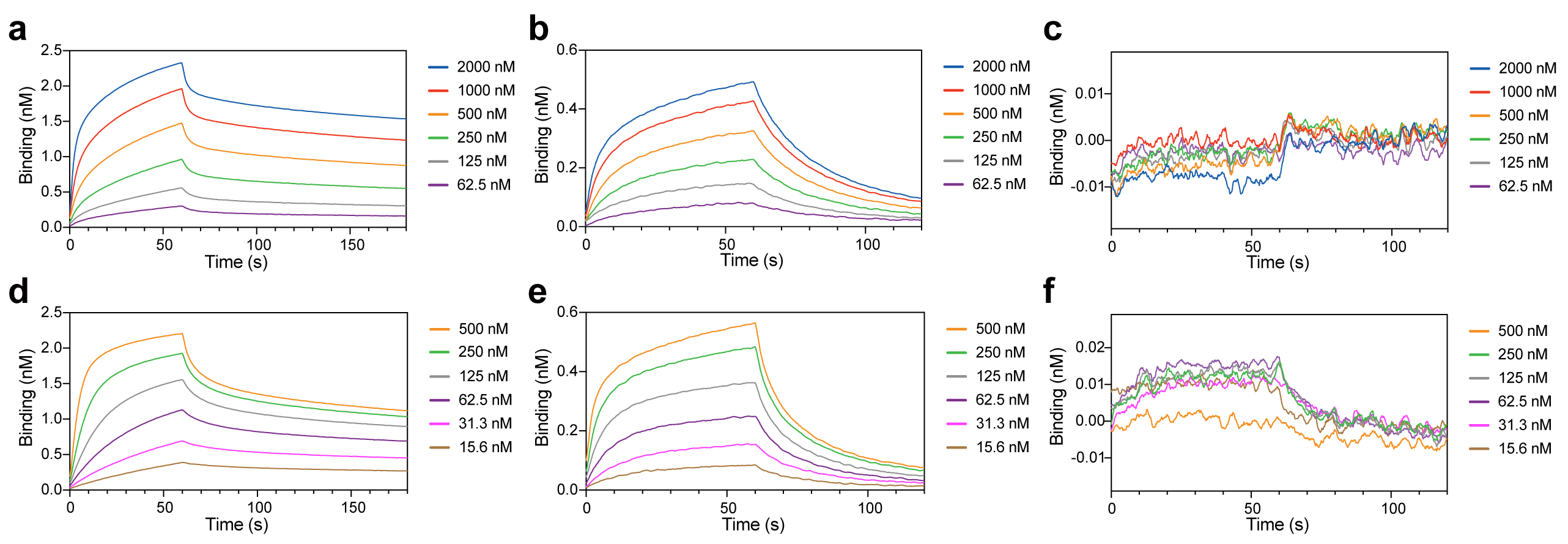


Figure. S5.

**Binding kinetics of TCRm Bi-NbTE determined by biolayer interferometry.** (a-c) Streptavidin biosensor coated with (a) biotinylated human CD3ε protein, (b) biotinylated human HLA-A2/WT1_126-134_ complex protein or (c) mimetic peptide was incubated with increasing concentrations of HLA-A2/WT1_126_ TCRm Bi-NbTE. (d-f) Streptavidin biosensor coated with (d) biotinylated human CD3ε protein, (e) biotinylated human HLA-A2/GPC3_144-152_ complex protein or (f) mimetic peptide was incubated with increasing concentrations of HLA-A2/GPC3_144_ TCRm Bi-NbTE. Association and dissociation phases are shown, with binding responses increasing in a concentration-dependent manner to both pMHC and CD3ε targets.


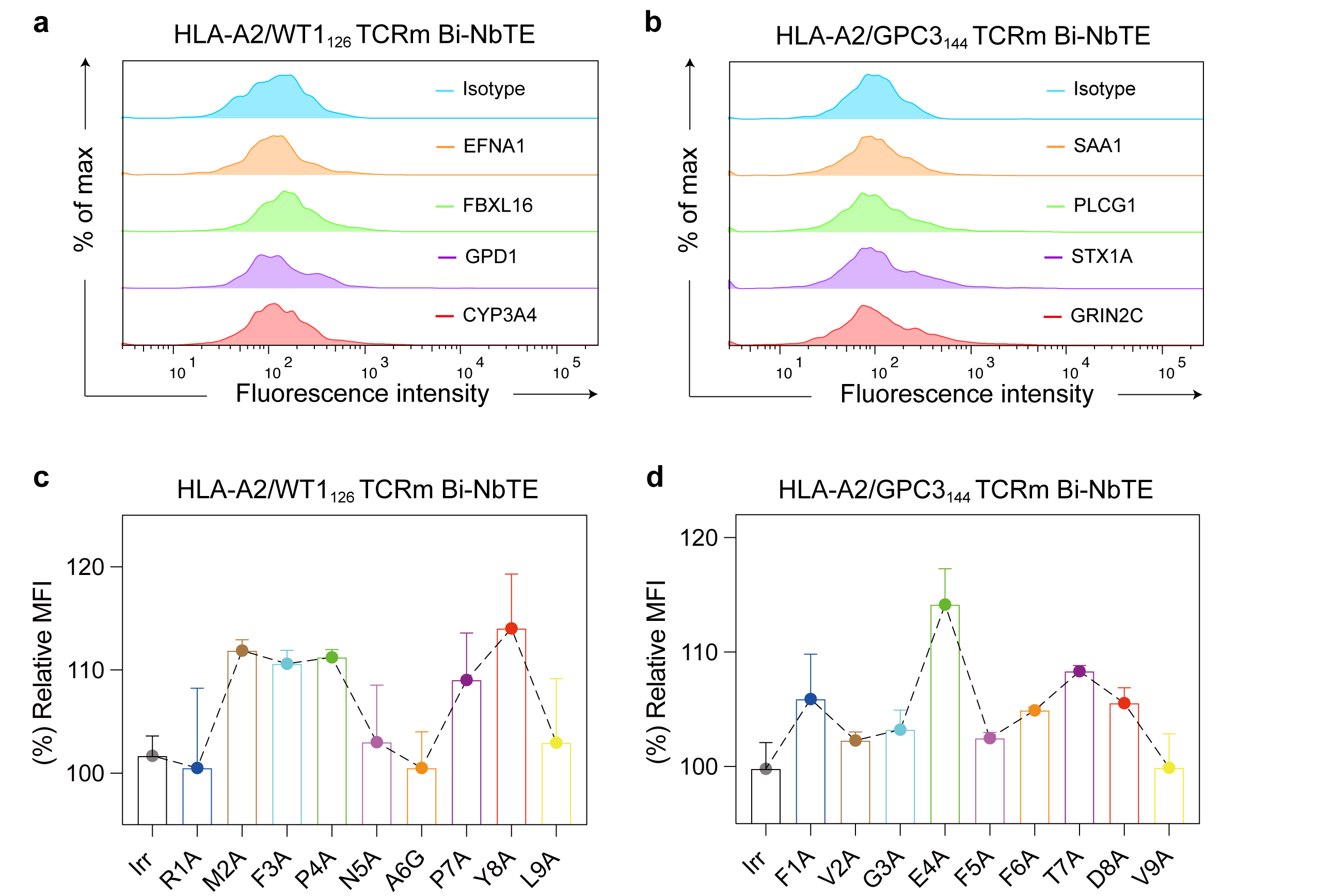


Figure. S6.

**Cross-reactivity profiling and epitope mapping of TCRm Bi-NbTE**. (a, b) Representative flow cytometry diagrams showing binding of (a) HLA-A2/WT1_126_ TCRm Bi-NbTE and (b) HLA-A2/GPC3_144_ TCRm Bi-NbTE to T2 cells pulsed with top-ranked predicted cross-reactive peptides identified by the sCRAP algorithm. No significant off-target binding was detected. (c, d) Alanine scanning mutagenesis and flow cytometry analysis reveals critical contact residues within the (c) RMFPNAPYL and (d) FVGEFFTDV epitopes for respective TCRm Bi-NbTE recognition. Substitutions at Arg1, Asn5, Ala6, and Leu9 significantly diminished HLA-A2/WT1_126_ TCRm Bi-NbTE binding, and residues Val2, Gly3, Phe5, and Val9 are particularly important for recognition by HLA-A2/GPC3_144_ TCRm Bi-NbTE.


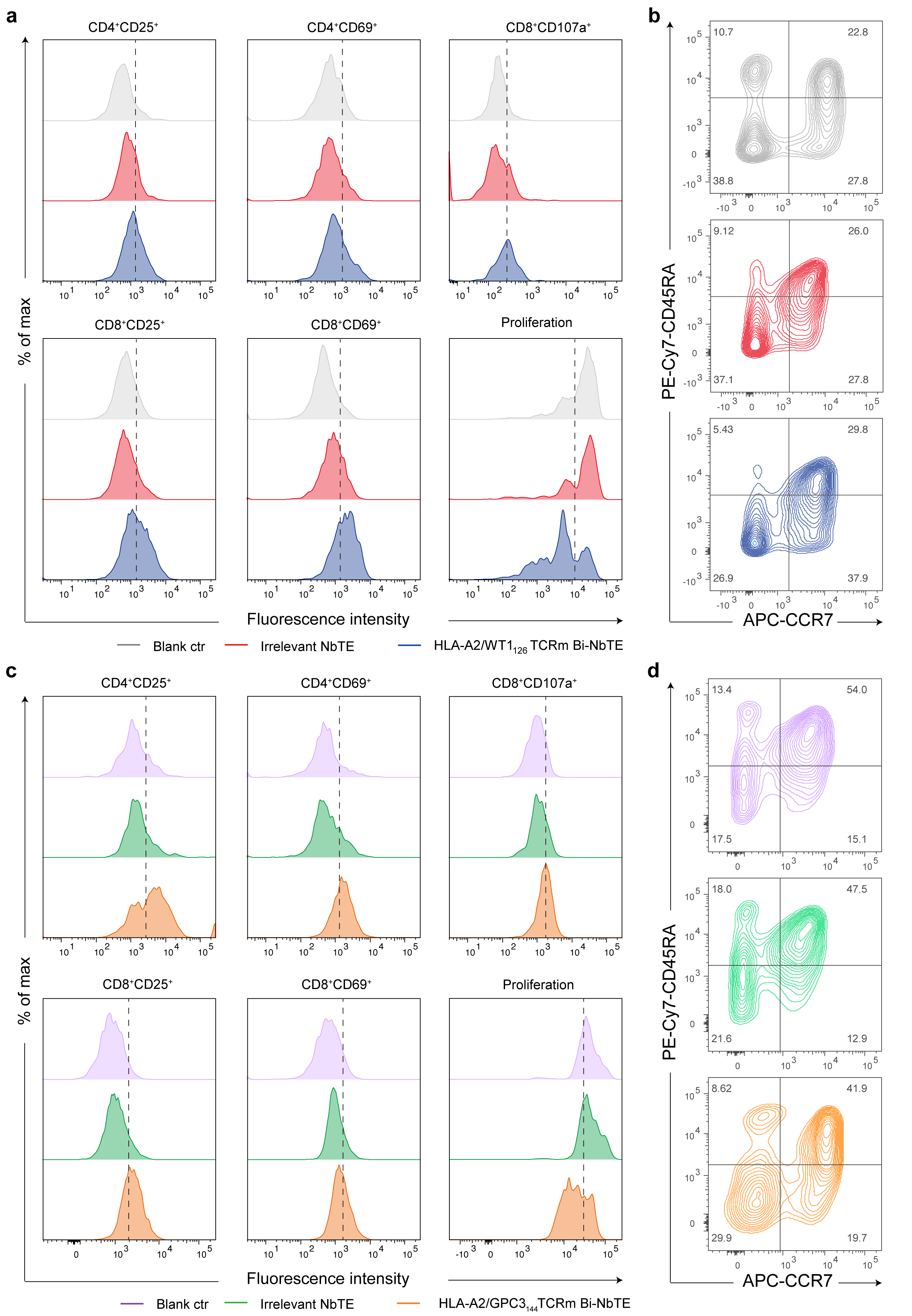


Figure. S7.

**Representative flow cytometry profiles of T cell activation and memory phenotypes.** (a, c) Overlaid histograms showing the expression levels of CD25, CD69, and CD107a, and histograms of CFSE dilution in T cells co-cultured with target cells in the presence of (a) HLA-A2/WT1_126_ TCRm Bi-NbTE or (c) HLA-A2/GPC3_144_ TCRm Bi-NbTE, compared to the Irrelevant NbTE or Blank ctr. (b, d) Representative histograms displaying CCR7 and CD45RA expression profiles to evaluate memory T cell subsets in the presence of (b) HLA-A2/ WT1_126_ TCRm Bi-NbTE or (d) HLA-A2/GPC3_144_ TCRm Bi-NbTE.


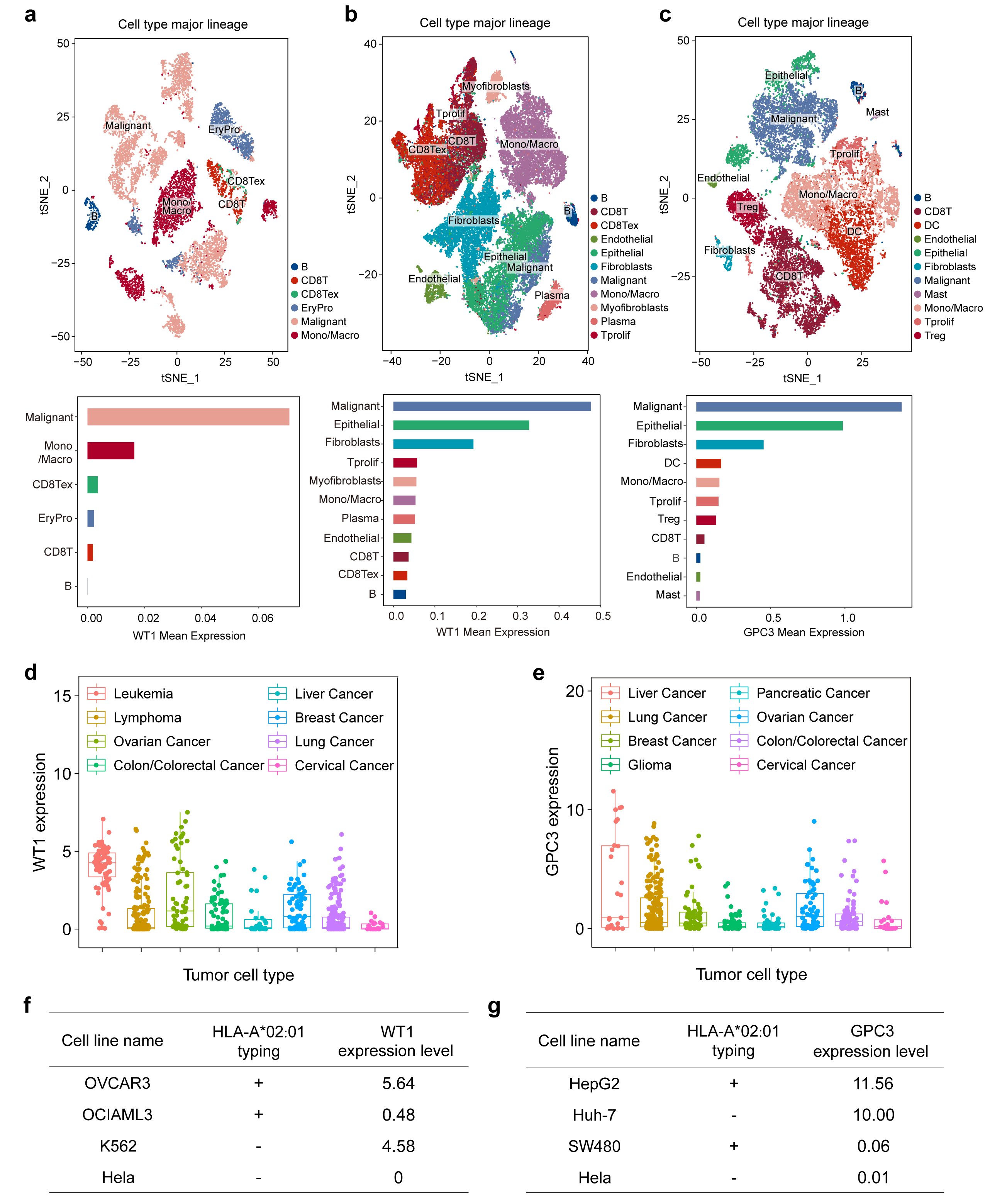


Figure. S8.

**Analysis of WT1 and GPC3 expression in public datasets.** (a, b) t-SNE visualization of single-cell RNA-seq data from acute myeloid leukemia (GSE154109) and ovarian cancer (GSE154600), with a bar chart quantifying WT1 expression level across distinct cell clusters. (c) t-SNE visualization of single-cell RNA-seq data from liver hepatocellular carcinoma (GSE166635), with a bar chart quantifying GPC3 expression level across distinct cell clusters. (d, f) WT1 expression across cell lines derived from CCLE database. (e, g) GPC3 expression across cell lines derived from CCLE database.


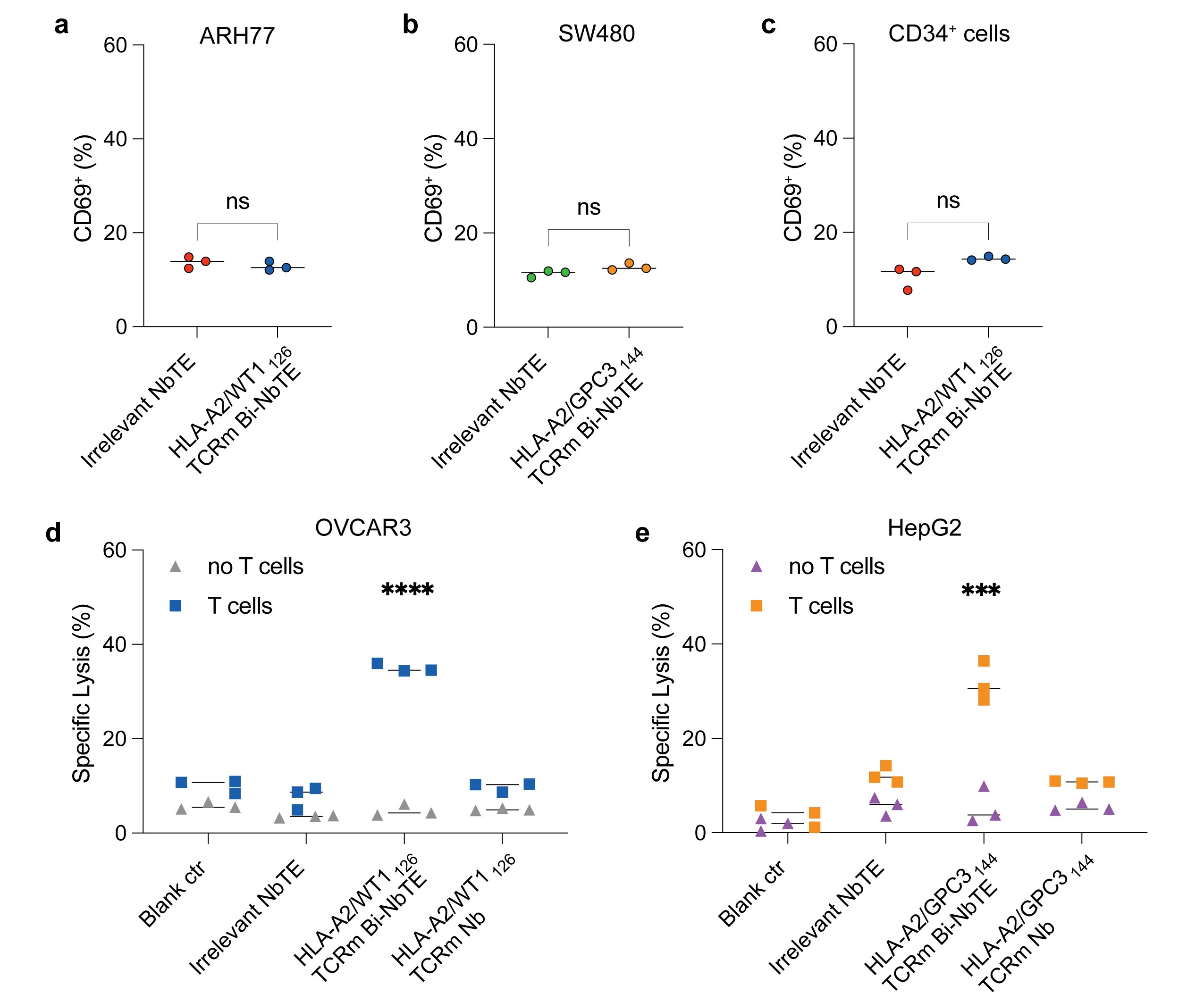


Figure. S9.

**Antigen-specificity and T cell-dependent cytotoxicity mediated by TCRm Bi-NbTE.** (a-c) T cell activation assays using (a) ARH77 (HLA-A2^+^/WT1^-^), (b) SW480 (HLA-A2^+^/GPC3^-^), (c) CD34^+^ cells coculured with T cells in the presence of indicated TCRm Bi-NbTE. No significant T cell activation was observed. (d, e) Cytotoxicity assays were performed on (d) OVCAR3 and (e) HepG2 cells in the presence or absence of T cells, treated with indicated TCRm Bi-NbTE, the corresponding TCRm Nb or other control molecules. Specific lysis was observed in the presence of both T cells and the corresponding TCRm Bi-NbTE, confirming T cell–dependent cytotoxicity. ****P* < 0.001; *****P* < 0.0001; ns not significant.


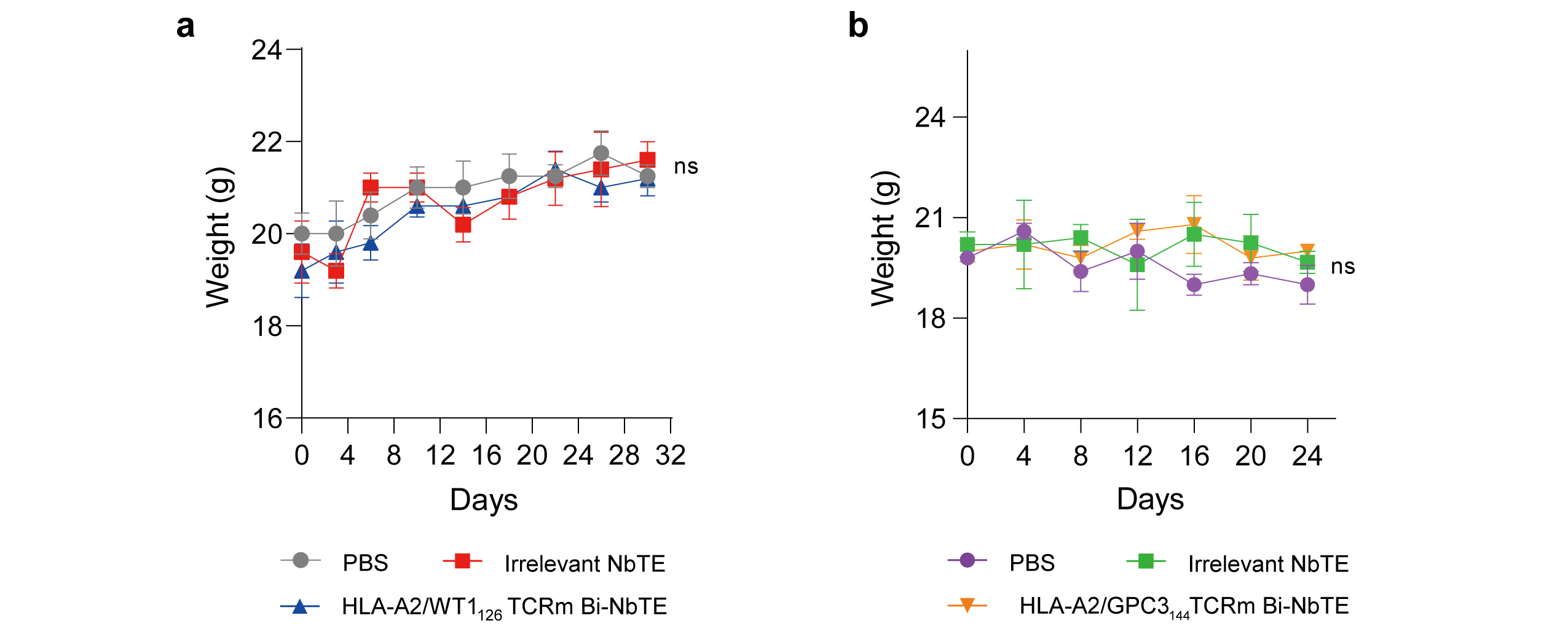


Figure. S10.

**Body weight monitoring in mouse xenograft models** **during TCRm Bi-NbTE treatment.** (a, b) Body weight curves of mice bearing (a) OVCAR3 tumors or (b) PDX tumors over the treatment period. No significant weight loss was observed in any treatment group, ns not significant.


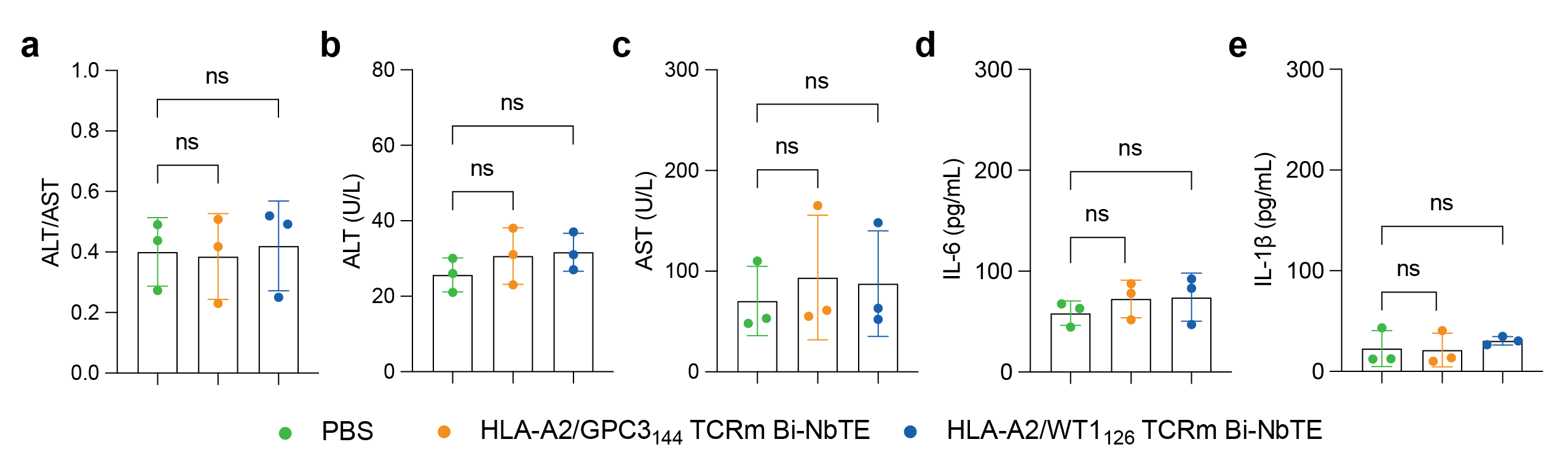


Figure. S11.

**Assessment of treatment-related adverse effects via serum analysis.** (a-c) Serum biochemical analysis of (a) ALT/AST·ratios, (b) ALT levels, and (c) AST levels in mice treated with TCRm Bi-NbTE or controls. (d, e) Serum cytokine analysis of (d) IL-6 and (e) IL-1β levels, suggesting minimal cytokine release risk, ns not significant.


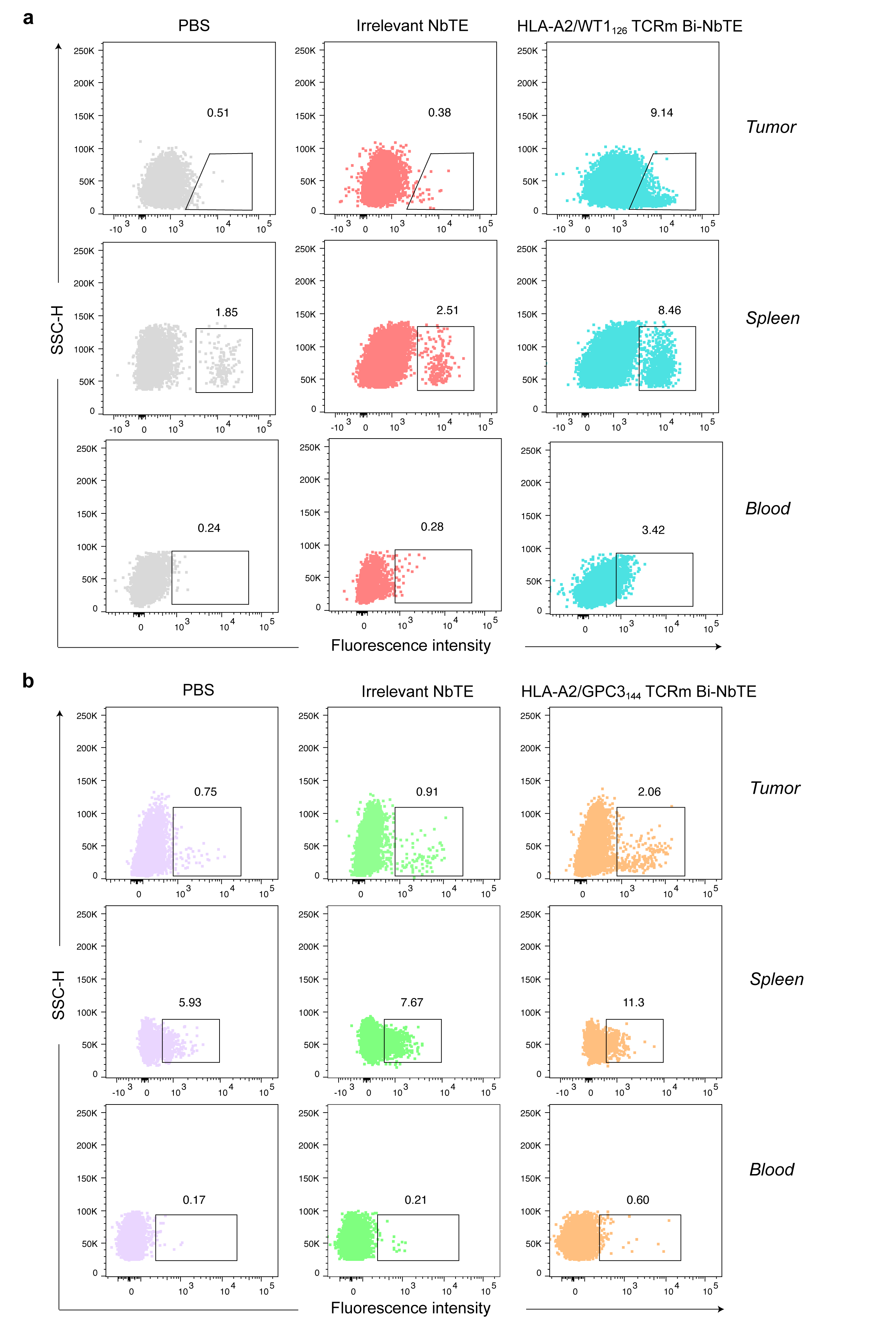


Figure. S12.

**Flow cytometry analysis of T cell infiltration after TCRm Bi-NbTE treatment.** (a, b) Representative flow cytometry plots showing CD3⁺ T cell populations in tumor tissues, spleens, and peripheral blood of mice treated with (a) HLA-A2/WT1_126_ TCRm Bi-NbTE or (b) HLA-A2/GPC3_144_ TCRm Bi-NbTE. Data demonstrate increased T cell infiltration and distribution, supporting the role of TCRm Bi-NbTE in promoting T cell recruitment and expansion *in vivo*.
